# Genome-wide characterization of histone deacetylases in *Fusarium proliferatum*: phylogeny, structure, and stress responses

**DOI:** 10.3389/fmicb.2025.1692364

**Published:** 2026-01-21

**Authors:** Hong-Mei Shi, Hong-Xin Liao, Jin-Rui Wen, Huan-Qi Cun, Yun-Ju Hong, Zhang-Feng Hu, Fu-Rong Xu, Sulukkana Noiprasert, Kanyaphat Apiwongsrichai, Xiao-Yun Liu, Xian Dong

**Affiliations:** 1 Yunnan Key Laboratory of Southern Medicinal Utilization, Yunnan University of Chinese Medicine, Kunming, China; 2 College of Life Sciences, Hubei Engineering Research Center for Protection and Utilization of Special Biological Resources in the Hanjiang River Basin, Jianghan University, Wuhan, China; 3 Key Laboratory of Soybean Disease and Pest Control (Ministry of Agriculture and Rural Affairs), Nanjing Agricultural University, Nanjing, Jiangsu, China; 4 School of Integrative Medicine, Mae Fah Luang University, Chiang Rai, Thailand

**Keywords:** histone deacetylases (HDACs), *Fusarium proliferatum*, gene family evolution, abiotic stress response, epigenetic regulation

## Abstract

**Background:**

*Fusarium proliferatum*, a globally distributed phytopathogen causing destructive root rot in economically vital crops, employs epigenetic mechanisms to adapt to environmental conditions.

**Results:**

Our genome-wide characterization identified eight histone deacetylase (*FpHDACs*) genes phylogenetically classified into RPD3/HDA1 and Sirtuin subfamilies. Comprehensive genomic characterization revealed two distinctive features: expanded domain architectures exemplified by the Arb2domain within *Fp_HDA1*, and subcellular localization prediction indicates—where most *FpHDACs* reside cytoplasmically under neutral pH, but undergo nuclear translocation in alkaline environments. Evolutionary diversification occurred principally via subfunctionalization rather than gene duplication, evidenced by non-clustered chromosomal distribution (8 genes across 5 chromosomes), divergent gene architectures in intron-exon organization and CDS lengths, and promoter *cis*-element enrichment featuring combinatorial stress-responsive signatures, most notably the dehydration-responsive DRE motifs exclusive to *Fp_HOS3*. Expression profiling analysis reveals a conserved global suppression of *FpHDACs* under abiotic stress, which is markedly potentiated by histone deacetylase inhibitor treatment. Crucially, the observed suppression was counterbalanced by a context-dependent induction of *Fp_HOS3*—specifically triggered under oxidative and cell wall stress, but not by other stressors. This specialized isoform functions as a compensatory epigenetic modulator, fine-tuning stress responses through targeted histone modification.

**Conclusion:**

This study provides the first systematic elucidation of the HDAC gene family’s core structural and functional characteristics in *F. proliferatum*, yielding novel insights into the adaptive strategies—both conserved and innovative—that underpin fungal stress epigenetics.

## 1 Introduction

The sustainable cultivation of medicinal herbs—including *Codonopsis pilosula* (Li et al., 2025) and *Astragalus membranaceus* (Li et al., 2021)—faces mounting pressure from root rot epidemics fueled by continuous cropping and fertilizer overuse (Wang et al., 2023; Wu et al., 2023). This devastating soil-borne disease, primarily caused by *Fusarium* pathogens, reduces yields and lowers medicinal quality (Han et al., 2025). Among these pathogens, the polyphagous fungus *Fusarium proliferatum* emerges as a particularly insidious threat. With global distribution and broad host range spanning *Salvia miltiorrhiza* (Jin et al., 2020) and *Panax notoginseng* (Zhang et al., 2025), it induces wilting and root rot while disseminating via contaminated seeds and (Shih et al., 2025) crop residues (Justyna et al., 2023). Compounding its impact, *F. proliferatum* also produces mycotoxins such as fumonisins and moniliformin, which further jeopardize the safety of medicinal products (Perincherry et al., 2019). Interestingly, studies in model plants like rice have revealed that epigenetic and transcriptional regulators—such as OsNLP6—play critical roles in mediating plant responses to abiotic stress and nutrient availability (Xin et al., 2025), suggesting that similar regulatory mechanisms may underpin stress adaptation in medicinal species.

Current management of *F. proliferatum* relies predominantly on broad-spectrum chemical fungicides, an approach increasingly constrained by environmental persistence, fungicide resistance, and non-target toxicity (Zeng et al., 2021). These limitations intensify the demand for sustainable, eco-friendly alternatives that target pathogen-specific vulnerabilities. Notably, emerging green antifungals—including plant-derived terpenoids like β-caryophyllene oxide (Liao et al., 2024) and menthone (Zhang et al., 2025) —exert their biocidal activity through interference with histone acetylation processes (Chang et al., 2016). These compounds selectively disrupt histone acetylation homeostasis, a conserved regulatory axis governing fungal development and virulence. By activating histone deacetylases (HDACs), they dysregulate acetylation-dependent transcriptional programs essential for pathogenicity (Wen et al., 2025). This mechanistic convergence positions HDACs as master regulators of epigenetic signaling in fungi, revealing a critical therapeutic vulnerability: precise modulation of HDACs activity can cripple stress adaptation, secondary metabolism, and host invasion mechanisms (Du et al., 2023). Despite their validated role as antifungal targets in models like *Aspergillus nidulans* (Pidroni et al., 2018) and *Ustilaginoidea virens* (Wang et al., 2024), the structural diversity, functional partitioning, and stress-responsive regulation of HDACs in *F. proliferatum* remain uncharacterized—a knowledge gap impeding the rational design of next-generation epigenetic antifungals against this agronomically devastating pathogen (Gong et al., 2024).

Histone acetylation, one of the most abundant modifications, is coordinately regulated by histone acetyltransferases (HATs) and HDACs: HATs add acetyl groups, whereas HDACs are responsible for removing these acetyl groups (Wang et al., 2024). Current research has established the antagonistic interplay between HATs and HDACs as a central epigenetic regulatory mechanism (Dubey et al., 2019). HATs generally promote gene activation, whereas HDACs suppress gene expression. In *Arabidopsis thaliana*, for instance, HDA6 reduces the acetylation level of WRKY63, while HAG1 markedly enhances it, illustrating how acetylation homeostasis is maintained through such antagonism (Shih et al., 2025). It remains to be explored whether HATs and HDACs in *F. proliferatum* regulate gene transcription and expression via a similar antagonistic mechanism. HDACs classified by cofactor dependence into Zn^2+^-dependent families (RPD3/HDA1/HD2) and NAD^+^-dependent sirtuins (SIR2), these enzymes orchestrate biological processes extending far beyond gene silencing—including cell differentiation, stress adaptation, and crucially, pathogenesis across diverse organisms (Lai et al., 2022). In plant-pathogenic fungi, HDACs emerge as pivotal virulence determinants, where their dysfunction directly compromises infection mechanisms: In *Magnaporthe oryzae*, *MoRpd3* and *MoHst4* differentially regulate mycelial growth, asexual development, and pathogenicity (Lin et al., 2021), while *Candida albicans* Sir2 mutants fail to express hyphal-specific genes—a critical deficiency that cripples morphological transition and pathogenicity (Zhao et al., 2021). Moreover, HDACs are key sentinels in environmental stress response. *Gossypium hirsutum* HDACs exhibit differential expression under drought and cold (Imran et al., 2020), while *Aspergillus flavus* SirE integrates osmotic and cell wall integrity signaling (Wen et al., 2022). This functional conservation underscores HDACs as central stress-adaptation nodes—yet in the high-impact pathogen *F. proliferatum*, their repertoire remains uncharted. No systematic study has explored its HDAC family’s structure, evolution, or roles in abiotic stress tolerance.

To bridge this critical knowledge gap, we conducted the first genome-wide characterization of HDACs genes in *F. proliferatum*, systematically identifying and phylogenetically classifying eight *FpHDACs* genes. We deciphered their structural evolution through synteny, motif distribution, and promoter *cis*-element analyses, mapped their expression dynamics under salt, oxidative, osmotic, and cell wall stress conditions, and correlated these molecular features with morphological phenotypes. Our work uncovers how *FpHDACs* architect stress adaptation in this pathogen—delivering mechanistic insights into fungal epigenetic regulation, while revealing druggable targets for next-generation antifungals.

## 2 Materials and methods

### 2.1 Fungal strain and culture conditions

The fungal strain used in this study was isolated from the root samples of *Panax notoginseng* infected with root rot disease. After isolation and purification, the strain was inoculated onto potato dextrose agar (PDA) medium and cultured at a constant temperature of 28 °C for 7 days. Subsequently, genomic DNA was extracted from the target strain for sequencing (Nie et al., 2024), and the sequencing results have been deposited in the GenBank database (accession number is OP430570.1). Sequence alignment analysis based on NCBI BLAST revealed that the obtained sequence exhibited 100% homology with *F. proliferatum* (MH712158.1).

### 2.2 Identification and physicochemical analysis of HDACs

The HDACs protein sequences from *F. proliferatum* (Taxonomy ID: 1227346), *Saccharomyces cerevisiae* (Taxonomy ID: 559292), *Homo sapiens* (Taxonomy ID: 9606), and *Arabidopsis thaliana* (Taxonomy ID: 3702) were retrieved from UniProt^1^ and NCBI^2^. Candidate HDACs were screened using NCBI CDD and InterPro^3^ (Blum et al., 2025) to verify deacetylase domains (E-value < 1.0). *FpHDACs* Physicochemical properties (isoelectric point, molecular weight, amino acid length) were predicted using ExPASy ProtParam^4^. Subcellular localization was inferred via WoLF PSORT^5^.

### 2.3 Phylogenetic reconstruction

Comparative analysis of HDAC sequences across *F. proliferatum*, *Saccharomyces cerevisiae*, *Homo sapiens*, and *Arabidopsis thaliana* employed ClustalW-aligned datasets for phylogenetic reconstruction. Unrooted trees were generated in MEGA11.0 using the Maximum likelihood (ML) method with 1,000 bootstrap replicates (Tamura et al., 2021). The generated phylogenetic tree file was imported into the online platform ChiPlot^6^ for graphical display and aesthetic optimization (Xie et al., 2023).

### 2.4 Protein domain architecture, phylogenetic reconstruction, motifs analysis, and gene structure

The conserved domains of HDACs proteins were identified using NCBI CDD. The identified domain information was further verified with UniProt (see footnote 1), and the verified conserved domains were visualized using TBtools software. MEGA 11.0 was used to construct a phylogenetic tree for analyzing the homology of *FpHDAC*s. The online program MEME^7^ was employed to detect the conserved motifs of HDACs proteins in *F. proliferatum*, with the number of predicted motifs set to 10. The GTF file was imported into the Gene Structure^8^ online tool integrated in TBtools software to analyze and visualize the exon-intron structure (Chen et al., 2020).

### 2.5 Chromosomal localization and synteny analysis

The physical locations of *FpHDACs* genes on chromosomes were determined based on the genome annotation GTF file (GCF_900067095.1) of *F. proliferatum* obtained from NCBI. The localization information was visualized using TBtools software. The GFF3 file and genome FASTA file (GCF_900067095.1) of *F. proliferatum* were retrieved from the Ensembl Fungi database^9^. The Circos and MCscan program was used to analyze gene duplication events in the *F. proliferatum* genome and the homology between the *FpHDACs* gene family and other selected species (*Fusarium oxysporum*, *Fusarium solani*, *Fusarium graminearum*, *Fusarium verticillioides, Magnaporthe oryzae*).

### 2.6 Protein tertiary structure and interaction networks

The three-dimensional structure data files of the target HDACs proteins were downloaded from the UniProt database and rendered in PyMOL (Schiffrin et al., 2020). Protein–protein interaction networks. Were predicted using STRING (confidence score > 0.4) and visualized in Cytoscape (version 3.10.1).

### 2.7 Promoter *cis*-elements analysis

Promoter regions (2,000 bp upstream of transcription start sites) were extracted from genome assemblies using TBtools. *Cis*-regulatory elements were predicted using PlantCARE (Lescot et al., 2002) and visualized with TBtools and Adobe Illustrator software was used to optimize the layout and enhance the aesthetics of the visualized maps.

### 2.8 Phenotypic analysis under abiotic stress

Phenotypic responses to abiotic stressors were assessed by culturing *F. proliferatum* strains on PDA supplemented with: salt stress (KCL: 1.0, 1.5, 2.0, and 2.5 M) (Araújo et al., 2020), oxidative stress (H_2_O_2_: 24, 48, 72, and 96 mM) (Concepción et al., 2017), osmotic stress (sorbitol: 13.7, 16.5, 19.2, and 22.0 mM) (Hou et al., 2023), and cell wall stress (Congo red: 0.1, 0.5, 0.9, and 1.3 g/L) (Cai et al., 2024). Control plates contained unmodified PDA. Technical triplicates of each treatment were incubated at 28 °C for 7 days, after which colony morphologies were qualitatively documented and growth quantitatively assessed through the cross-streaking method. All phenotypic data were statistically analyzed using GraphPad Prism 10, and statistical significance analysis was performed using SPSS. Furthermore, hydrogen peroxide was added to the PDB medium to establish oxidative stress conditions at varying concentrations (24, 48, 72, and 96 mM). An equal volume of *F. proliferatum* spore suspension was simultaneously introduced to achieve a final concentration of 1 × 10^7^ spores/mL. The samples were subsequently subjected to filtration after 12, 24, and 48 h of incubation.

### 2.9 Effect of HDAC inhibitors on stress tolerance in *F. proliferatum*

In the preliminary experiment, Trichostatin A (TSA) and Niacinamide (Nic) solutions were added to standard PDA medium to establish treatment groups with varying concentrations (Brandão et al., 2015; Xue et al., 2023). Solvent controls and blank controls were included, and all treatments were performed in triplicate. After 4 days of incubation, colony diameters were measured, and mycelia were harvested for qRT-PCR analysis to screen for optimal concentrations. Based on the pre-experimental screening, 1.5 μM TSA (dissolved in DMSO) and 30 μM Nic (dissolved in water) were selected as HDAC inhibitor treatment groups. Intermediate concentrations from four abiotic stress treatment groups were chosen (salt stress: 1.5 M; oxidative stress: 72 mM; osmotic stress: 16.5 mM; cell wall inhibitor: 0.5 g/L). The core experimental groups consisted of media supplemented with both HDAC inhibitors and abiotic stress agents, with stress-only treatment groups serving as controls. *F. proliferatum* was cultured on PDA medium containing the aforementioned treatments, along with solvent controls (DMSO) and blank controls (CK), all in triplicate. After 7 days of incubation, colony diameters were measured using the cross-cross method.

### 2.10 RNA extraction and real-time quantitative polymerase chain reaction

After being cultured for 7 days in PDA medium supplemented with stress-inducing agents (at concentrations consistent with those detailed in Sections 2.8 and 2.9), the mycelia were rapidly frozen in liquid nitrogen and ground. Total RNA was extracted using Steadypure Plant RNA Kit (Accurate Biotechnology Co. Ltd.), followed by DNase I treatment. RNA integrity was confirmed via 1.5% MOPS-formaldehyde gels. cDNA was synthesized from 1 μg RNA using Evo M-MLV RT Kit (AG11728). qRT-PCR was performed on QuantStudio 5 (Thermo Fisher Scientific) with Taq SYBR Green qPCR Premix (EG20117M) under: 95 °C/30s; 40 cycles of 95 °C/5 s, 60 °C/30s. QTUB (β-tubulin) served as the reference gene. Primers are listed in Supplementary Table S1. Relative expression was calculated by 2^−ΔΔCt^ with three biological and technical replicates (Liu et al., 2023). Data visualization used TBtools and Adobe Illustrator.

## 3 Results

### 3.1 Genome-wide identification and physicochemical characterization of *FpHDACs*

Comprehensive screening of the *F. proliferatum* genome via NCBI and UniProt databases identified eight histone deacetylase (HDACs) genes, designated according to conserved protein domain architectures (Table 1). Physicochemical profiling using ProtParam revealed substantial diversity among *FpHDACs*: *FP_HOS3* emerged as the largest protein (1,165 amino acids; MW 125.81 KDa), while *FP_SIRT5* constituted the smallest (295 aa; MW 32.51 KDa). Analysis of the theoretical isoelectric points (pI) revealed a spectrum from acidic (4.57) to basic (9.79). Furthermore, subcellular localization predictions demonstrated a bipartite distribution (i.e., presence in both the nucleus and cytoplasm), correlating with the pI values: four proteins (pI < 6.5) were predicted for the cytoplasm, and four (pI > 6.5) for the nucleus. This compartmentalized pI partitioning likely reflects functional adaptation to organelle-specific microenvironments. Collectively, these data reveal extensive physicochemical divergence within the *F. proliferatum* HDACs gene family, suggesting evolutionary specialization among members.

**Table 1:** Comprehensive physicochemical profiling of *FpHDACs.*

Gene name	Gene ID	Localization	Protein length (aa)	Molecular weight (KDa)	pI
*Fp_HOS2*	FPRO_00584	Cytoplasmic	499	56.17	5.46
*Fp_HDA1*	FPRO_13046	Cytoplasmic	736	82.85	5.33
*Fp_HOS3*	FPRO_10610	Nuclear	1,165	125.81	9.40
*Fp_RPD3*	FPRO_01701	Cytoplasmic	649	72.10	4.57
*Fp_SIRT1*	FPRO_03139	Nuclear	487	54.44	6.41
*Fp_SIR2*	FPRO_08373	Nuclear	968	106.16	8.09
*Fp_HST4*	FPRO_01165	Nuclear	625	68.59	9.42
*Fp_SIRT5*	FPRO_03415	Cytoplasmic	295	32.51	5.82

### 3.2 Phylogenetic analysis of the *FpHDACs* gene family

**Figure 1:**
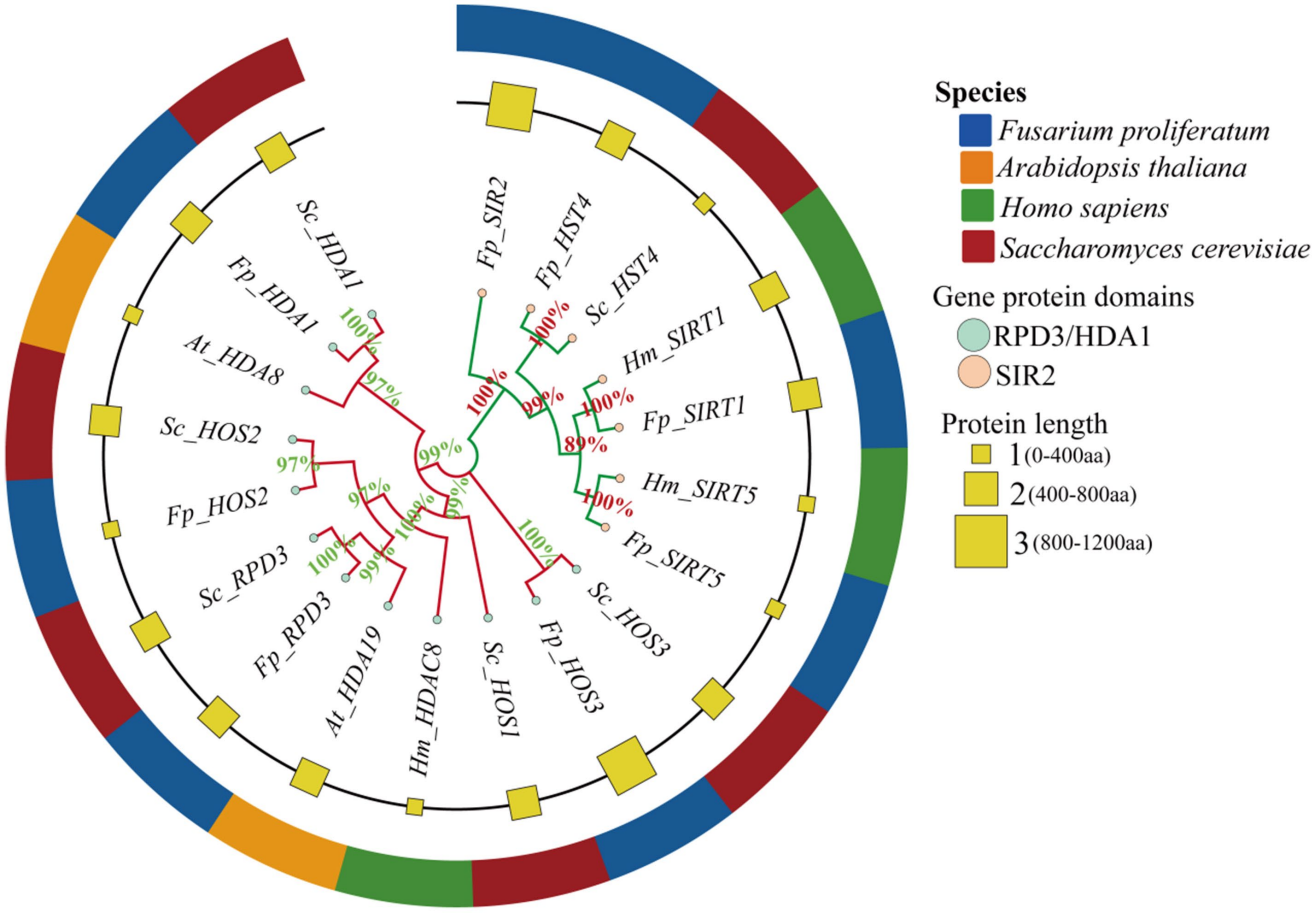
Phylogenetic tree of *HDACs* genes. The *HDACs* genes include those from *F. proliferatum*, *Saccharomyces cerevisiae*, *Homo sapiens*, and *Arabidopsis thaliana*, and the tree was constructed using the Maximum Likelihood (ML) method in MEGA11.0. In the figure, genes from different species are indicated by boxes of different colors (blue, red, green, and orange), where the blue boxes represent *F. proliferatum*, red boxes represent *Saccharomyces cerevisiae*, green boxes represent *Homo sapiens*, and orange boxes represent *Arabidopsis thaliana*. The size of the yellow color blocks indicates the protein length; “aa” stands for amino acid, where 1 represents a protein length ranging from 0 to 400 amino acids, 2 represents a length ranging from 400 to 800 amino acids, and 3 represents a length ranging from 800 to 1,200 amino acids. Cyan and orange circles represent protein domains, with cyan indicating proteins of the RPD3/HDA1 subfamily and orange indicating proteins of the SIR2 subfamily. The branches of the phylogenetic tree are represented by red and green lines, where red lines represent branches of the RPD3/HDA1 subfamily and green lines represent branches of the SIR2 subfamily. The red and green fonts represent the genetic affinity ratios among HDACs genes, and the black fonts indicate the names of HDACs genes from the four species.

To resolve the evolutionary relationships of *HDACs* in *F. proliferatum*, we constructed an unrooted phylogenetic tree using the Maximum Likelihood method in MEGA 11.0. The analysis incorporated HDAC protein sequences from *F. proliferatum*, *Saccharomyces cerevisiae*, *Homo sapiens*, and *Arabidopsis thaliana* (Figure 1). The eight *FpHDACs* unequivocally segregated into two phylogenetically distinct subfamilies: RPD3/HDA1 and SIR2. This bifurcation exhibited perfect concordance with conserved domain classifications. Within each clade, members demonstrated striking sequence conservation (89–100% homology), with several genes establishing unambiguous one-to-one orthology—notably *Fp_HST4*/*Sc_HST4* (SIR2 subfamily) and *Fp_HOS3*/*Sc_HOS3* (RPD3/HDA1 subfamily). Crucially, *F. proliferatum* HDACs displayed significantly closer evolutionary affinity to *Saccharomyces cerevisiae* orthologs. This indicates that the HDACs of these two groups of species may have originated from a common ancestor.

### 3.3 Structural diversification of *FpHDACs* proteins: domain architecture, conserved motifs, and gene organization

**Figure 2:**
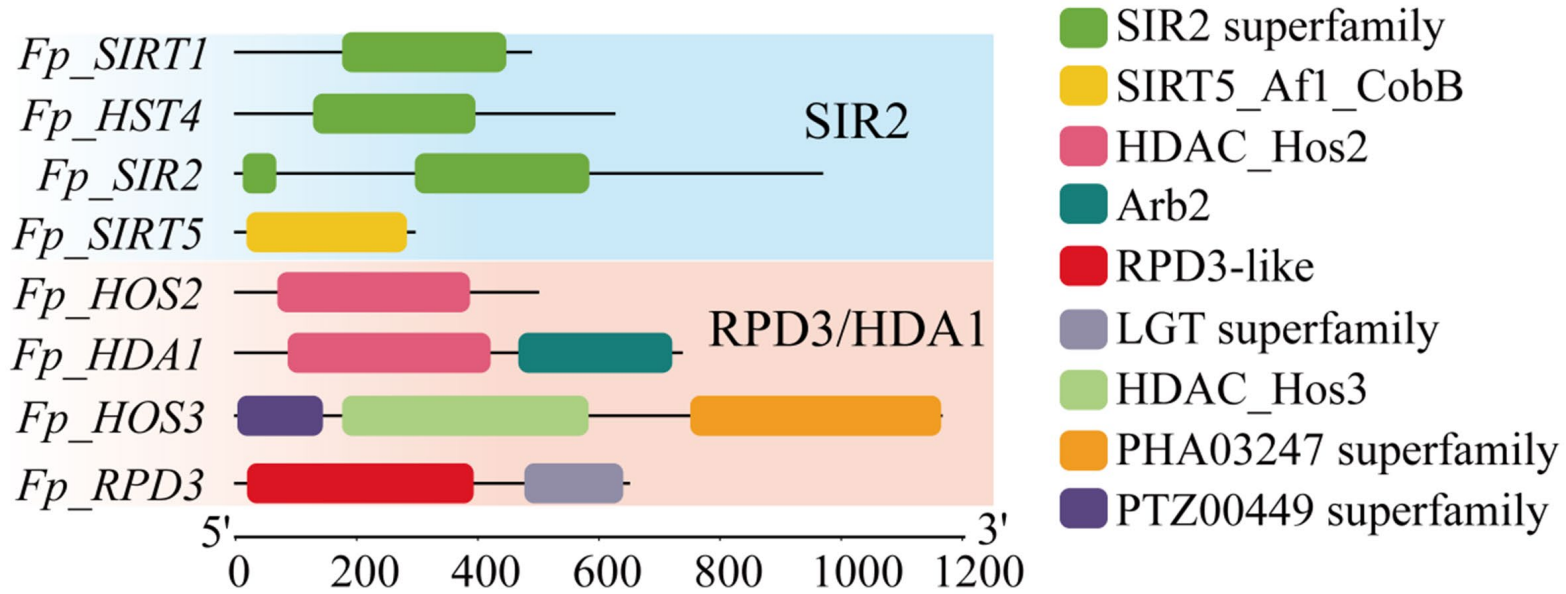
Conserved domains of *FpHDACs* proteins. In the figure, frames of different colors represent different conserved domains, with blue indicating the SIRT2 subfamily and orange indicating the RPD3/HDA1 subfamily.

Comprehensive domain analysis of the eight *FpHDACs* proteins revealed distinct subfamily-specific architectures (Figure 2). The SIR2 subfamily (*Fp_SIRT1*, *Fp_HST1, Fp_SIR2*, and *Fp_SIRT5*) exclusively contained canonical SIR2/SIRT5 domains, while RPD3/HDA1 members exhibited structural elaboration: Except for *Fp_HOS2*, other members of the RPD3/HDA1 subfamily (*Fp_HDA1*, *Fp_HOS3*, and *Fp_RPD3*) contain 1 to 2 additional domains. *Fp_HDA1* contained an auxiliary Arb2 domain, *Fp_HOS3* featured PHA03247 and PTZ00449 superfamily domains, and *Fp_RPD3* possessed an LGT superfamily domain—indicating potential cooperative roles in deacetylation regulation beyond core enzymatic functions.

**Figure 3:**
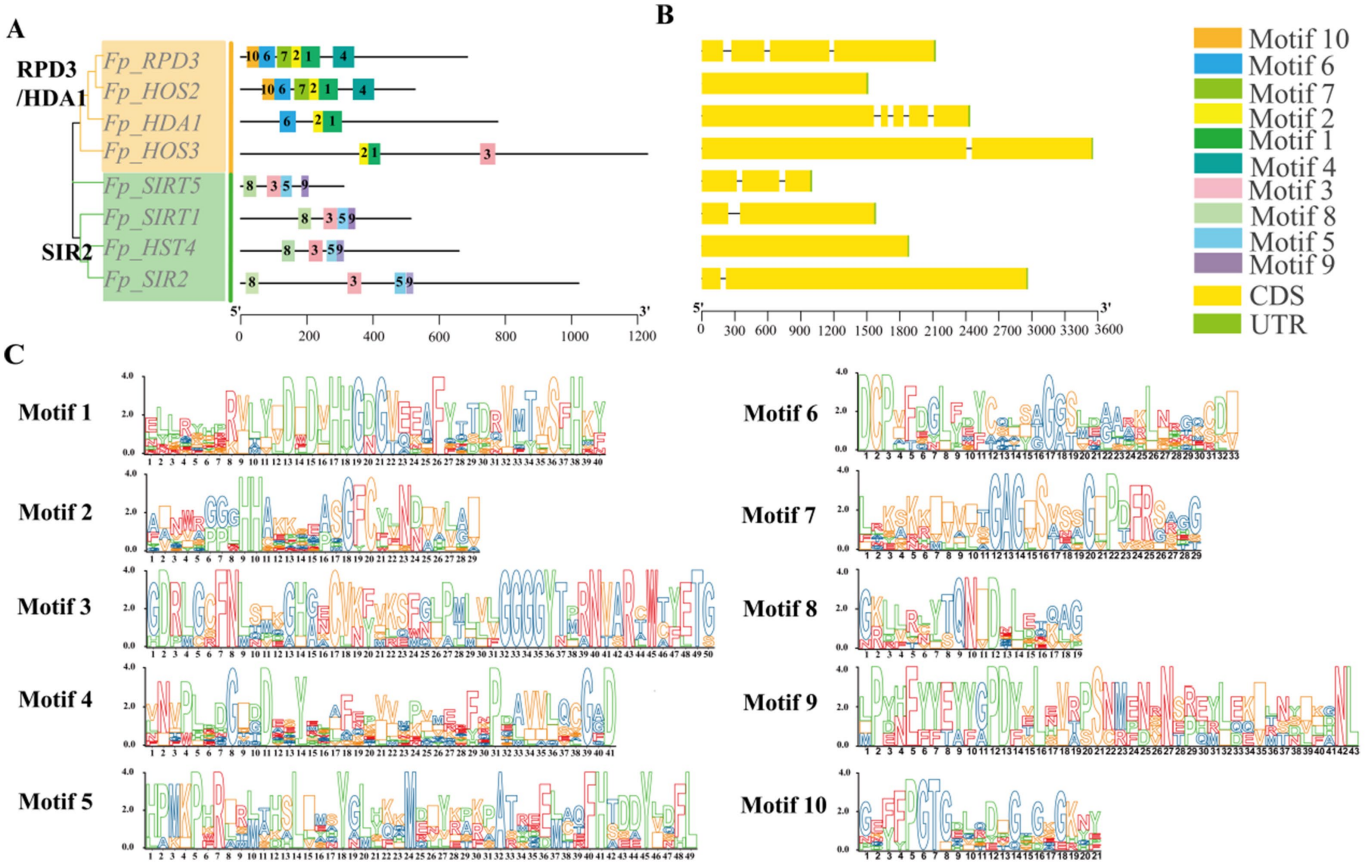
Phylogenetic relationships, structural conservation and diversity of *FpHDACs*. **(A)** Phylogenetic tree and conserved motifs analysis of *FpHDACs* genes. The phylogenetic tree was constructed using the Maximum Likelihood (ML) method, and the conserved motif architectures of *FpHDACs* proteins were predicted via MEME analysis. Boxes in different colors and black numbers represent different conserved motifs (1–10). The RPD3/HDA1 subfamily is highlighted in orange, and the SIR2 subfamily is highlighted in green. **(B)** Exon-intron organization of *FpHDACs* genes. Yellow rectangles denote exons connected by black lines representing introns. **(C)** Sequence conservation profiles of the 10 motifs visualized as sequence logos. Logo height (*y*-axis, bits) reflects positional conservation, while residue height within each stack indicates relative frequency at corresponding sequence positions (*x*-axis).

To investigate the evolutionary relationships of *FpHDACs* in *F. proliferatum*, we constructed an unrooted phylogenetic tree by aligning the sequences of 8 *FpHDACs* proteins. Based on the phylogenetic relationships of *FpHDACs* genes, we analyzed the conserved motifs and gene structures of *FpHDACs* genes (Figure 3). Conserved motif analysis via MEME identified 10 significant motifs (Figures 3A,C). All RPD3/HDA1 proteins maintained universal Motif 1–2, supplemented by Motif 6 in *Fp_RPD3/Fp_HOS2*/*Fp_HDA1* and unique Motif 3 in *Fp_HOS3*. Conversely, the SIR2 subfamily uniformly conserved Motifs 3, 5, 8, and 9. Gene structure examination revealed exceptional plasticity—exon counts ranged from one (*Fp_HOS2*) to five (*Fp_HDA1*), with RPD3/HDA1 members exhibiting substantially longer coding sequences than SIR2 counterparts. Remarkably, substantial intra-subfamily heterogeneity persisted, evidenced by *Fp_HOS2*’s single exon versus *Fp_HDA1*’s five exons within RPD3/HDA1. This collective evidence demonstrates profound structural diversification, where RPD3/HDA1 proteins evolved complex architectures with accessory domains while SIR2 members preserved streamlined structures—a divergence likely underpinning functional specialization in epigenetic control.

### 3.4 Chromosomal localization, gene duplication, and interspecies synteny of the *FpHDACs* gene family

**Figure 4:**
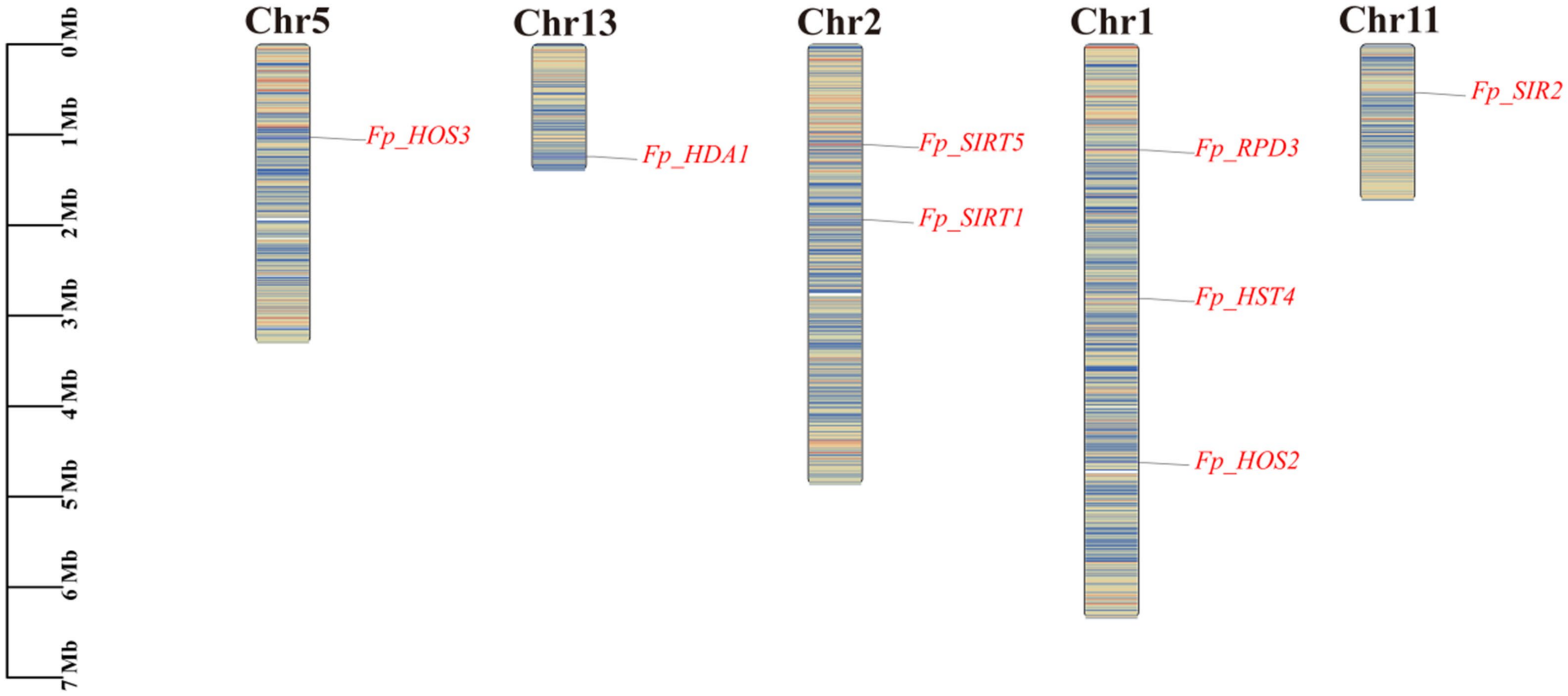
Chromosomal distribution and genomic context of *FpHDACs* genes. Physical mapping of eight *FpHDACs* genes across the *F. proliferatum* genome. Chromosome lengths (Mb) are indicated on the left vertical axis. *FpHDACs* loci are marked in red on the right side of each chromosome. Background colors represent regional gene density gradients, with red indicating high-density regions and blue denoting low-density zones.

Chromosomal mapping revealed an asymmetric distribution of the eight *FpHDACs* genes across five *F. proliferatum* chromosomes (Figure 4). Chromosome 1 harbored three genes (*Fp_RPD3*, *Fp_HST4*, *Fp_HOS2*), Chromosome 2 contained two (*Fp_SIRT5*, *Fp_SIRT1*), while Chromosomes 5, 11, and 13 each possessed one (*Fp_HOS3*, *Fp_SIR2*, and *Fp_HDA1* respectively). Notably, these genes exhibited dispersed genomic localization without evidence of tandem duplication clusters, though certain loci (*Fp_RPD3*, *Fp_SIRT5*) resided in gene-dense chromosomal regions.

**Figure 5:**
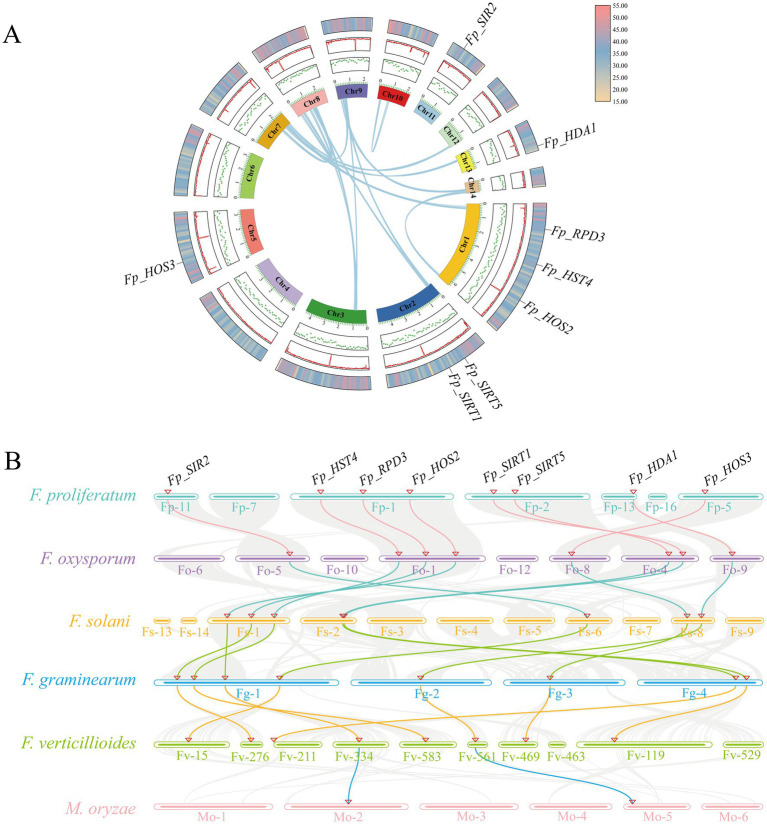
Intra- and inter-species synteny analysis of *FpHDACs* genes. **(A)** Intraspecific synteny analysis of *FpHDACs* genes. The innermost ring represents distinct chromosomes, with green dots indicating GC content variations in single-stranded DNA. The red ring denotes the GC ratio for each chromosome, while the outermost ring displays chromosome density (red: high-density regions; blue: medium-density regions; yellow: low-density regions). Blue background lines illustrate syntenic relationships, with chromosome circumference units marked in megabases (Mb). **(B)** Comparative synteny with five fungal pathogens. Gray lines represent background syntenic blocks between *F. proliferatum* (center) and *Fusarium oxysporum*, *Fusarium solani*, *Fusarium graminearum*, *Fusarium verticillioides*, and *Magnaporthe oryzae* (peripheral chromosomes). Gray background lines represent syntenic blocks of HDACs genomes, with syntenic HDACs gene pairs highlighted in colored lines. Chromosomes of different species are depicted as colored bars, with numerical identifiers positioned at the base. *FpHDACs* genes are annotated in black font.

Gene duplication is the core mechanism driving functional diversification and expansion of gene families (Yuan et al., 2015). To elucidate the evolutionary patterns of *FpHDACs* genes in *F. proliferatum*, this study systematically conducted intra- and interspecies synteny analyses by integrating the MCScanX algorithm with Circos visualization technology on the TBtools platform. In the intraspecies synteny analysis (Figure 5A), we observed that none of the eight identified *FpHDACs* genes exhibited evidence of paralogous gene duplication events (e.g., although *Fp_RPD3* and *Fp_HOS2* are physically adjacent on Chr1, they did not form a paralogous gene cluster). This suggests that the HDACs family in *F. proliferatum* did not evolve through gene duplication, and its genomic architecture is highly conserved and stable, with a fixed number of members throughout evolution. These findings imply that the functional diversity of *FpHDACs* genes likely arises from functional divergence, enabling individual members to execute specialized regulatory roles.

To further investigate the potential evolutionary processes of the HDACs gene family, we performed interspecies synteny analysis using *F. proliferatum*, *Fusarium oxysporum*, *Fusarium solani*, *Fusarium graminearum*, *Fusarium verticillioides*, and the ascomycete pathogen *Magnaporthe oryzae* (Figure 5B). The results revealed that all eight *FpHDACs* genes exhibited syntenic relationships with the four *Fusarium* species. However, only two genes (*Fp_HOS2* and *Fp_HDA1*) maintained synteny with *Magnaporthe oryzae*. Additionally, *Fp_SIRT1* and *Fp_SIRT5* were found to be associated with orthologous genes in *Fusarium solani*. The presence of highly conserved syntenic blocks between *F. proliferatum* and other *Fusarium* species supports their origin from common ancestral genes followed by species divergence. The limited synteny of *Fp_HOS2* and *Fp_HDA1* with *Magnaporthe oryzae* suggests significant evolutionary divergence between different genera within Ascomycota. In the absence of gene duplication, the HDAC family in *F. proliferatum* appears to have achieved functional specialization primarily through subfunctionalization—a process by which duplicated genes partition ancestral functions between them (Abubakar et al., 2023), highlighting the evolutionary plasticity of epigenetic regulatory networks.

### 3.5 Integrated structural and functional characterization of *FpHDACs*

**Figure 6:**
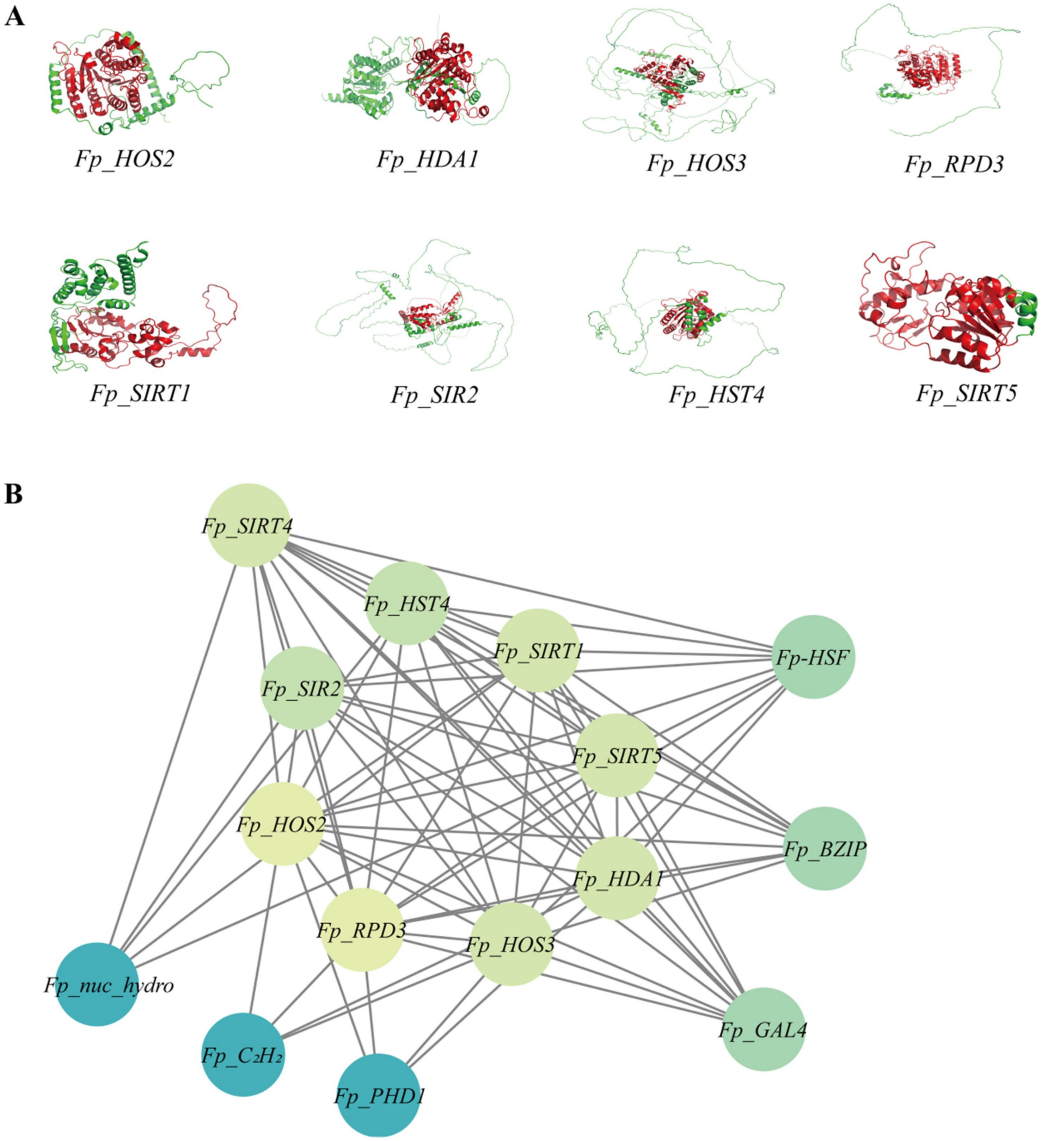
Three-dimensional structure and protein–protein interactions of *FpHDACs.* **(A)** The 3D structural model of HDACs proteins predicted through homology modeling using PyMOL software, with green representing the protein structure and red indicating the core protein region. **(B)** The protein–protein interaction (PPI) network of HDACs analyzed via Cytoscape software. Circles with varying colors correspond to ASPL values, gray lines denote PPI relationships, inner black labels represent *FpHDACs* proteins, while outer black labels indicate proteins interacting with *FpHDACs* genes.

To elucidate the structural foundations and regulatory networks of *FpHDACs*, we generated homology-based tertiary structures for all eight proteins using PyMOL, revealing distinct subfamily-specific spatial conformations (Figure 6A). Protein–protein interaction profiling via STRING identified a cohesive network comprising 15 nodes—the eight *FpHDACs* plus seven functional partners—exhibiting extensive cross-subfamily connectivity alongside intra-subfamily modules (Figure 6B). Strikingly, SIR2 and RPD3/HDA1 subfamilies formed numerous inter-group interactions while maintaining specialized internal connectivity patterns. Key interactors included stress-response regulator *Fp_HSF* (heat shock factor), transcriptional mediators *Fp_BZIP* (bZIP transcription factor) (Martignago et al., 2025), metabolic coordinator *Fp_GAL4* (galactose metabolism activator) (Johnston et al., 1986), and epigenetic modulator *Fp_SRT4* (NAD^+^-dependent deacetylase), all engaging multiple *FpHDACs*. Functional specialization was evident: nucleotide hydrolase *Fp_nuc_hydro* interacted exclusively with SIR2 members, while epigenetic regulators *Fp_PHD1* (demethylase activity modulator) and *Fp_C_2_H_2_* (Zinc finger transcription factor) showed RPD3/HDA1-specific binding. This interactome positions *FpHDACs* as multifunctional hubs integrating stress signaling (via *Fp_HSF*), transcriptional control (*Fp_BZIP*), epigenetic fine-tuning (*Fp_PHD1*), and nucleotide metabolism (*Fp_SRT4*) to orchestrate fungal development and environmental adaptation.

### 3.6 Comprehensive *cis*-regulatory landscape of *FpHDACs* promoters

**Figure 7:**
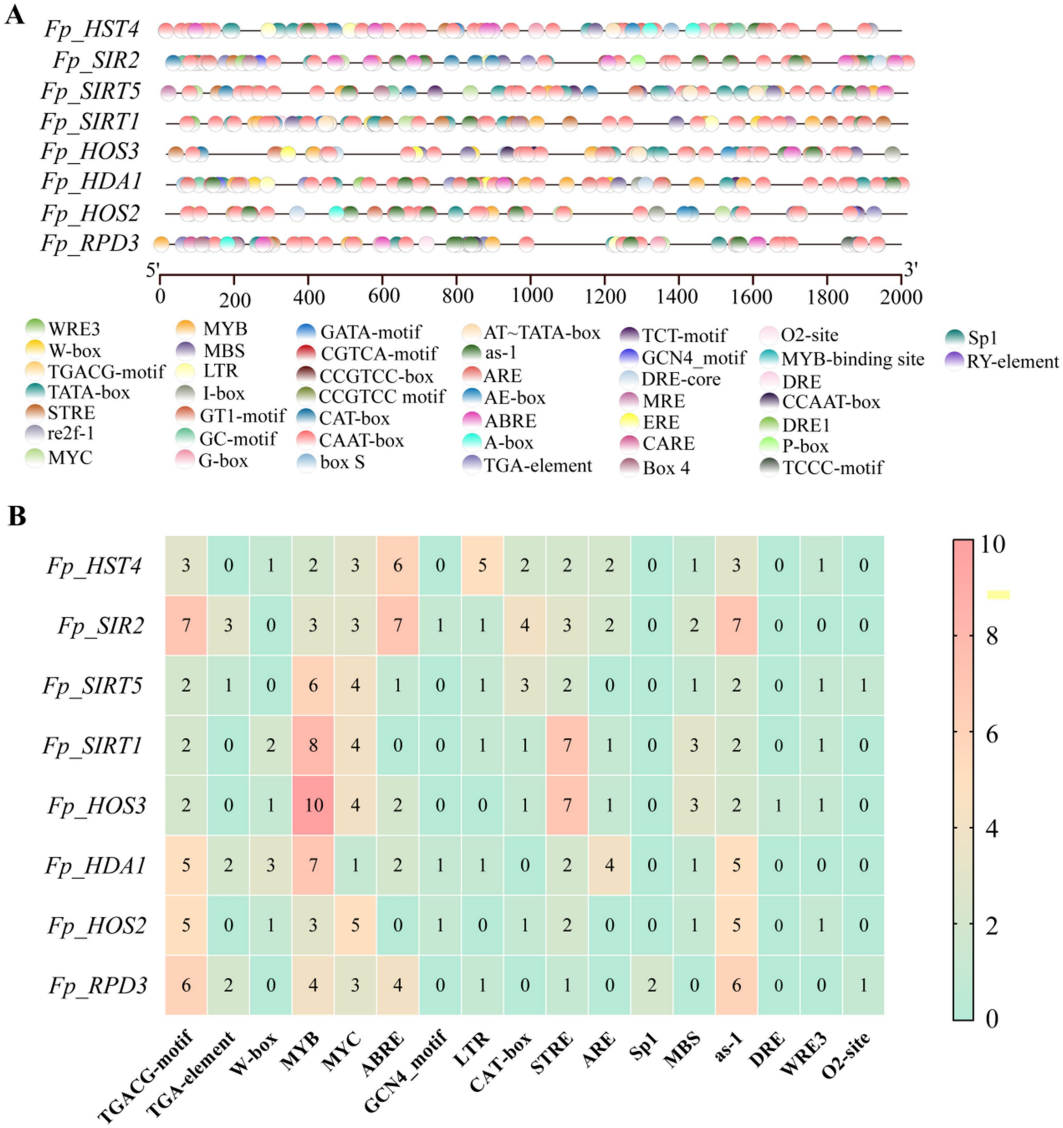
*Cis*-acting elements in *FpHDACs* gene promoters. **(A)** Schematic representation of all *cis*-acting elements in HDACs genes, with different colored circles denoting distinct types of *cis*-acting elements. **(B)** Heatmap analysis of abiotic stress-responsive cis-acting elements in HDACs genes, where escalating red intensity correlates with increasing element abundance, while green denotes absence of *cis*-regulatory elements. Numeric annotations indicate quantitative element counts.

Genome-wide profiling of promoter regions (2,000 bp upstream of transcription start sites) identified 44 distinct *cis*-regulatory elements across eight *FpHDACs* genes, via PlantCRE analysis (Figure 7A; full inventory in Supplementary Table S2). Core promoter elements (TATA/CAAT-boxes) were universally present alongside four functional categories: light-responsive (G-box, BOX-4, Sp1), hormone-responsive (MeJA, ABA, GA-related), stress-responsive (MYB, ARE, LTR), and developmental elements (CAT-box, RT-element). Light-response elements predominated quantitatively, with G-box and BOX-4 exhibiting broad distribution while Sp1 occurred exclusively in *Fp_RPD3*—indicating potential photoregulatory roles. Among hormone-response motifs, MeJA-related elements (CGTCA/TGACG) predominated, followed by ABRE (ABA-responsive). Stress-response analysis revealed ubiquitous drought-responsive MYB elements (2–10 copies/gene), maximally enriched in *Fp_HOS3* (10 copies) versus minimal in *Fp_HST4* (2 copy). Oxidative stress element as-1 was ubiquitous present, whereas drought-inducible DRE occurred exclusively in *Fp_HOS3*. Pathogen-responsive W-box appeared sporadically (1–3 copies in 5 genes), absent in three members (Figure 7B). Furthermore, developmental regulatory elements showed selective enrichment: endosperm-specific GCN4_motif localized *Fp_SIR2*/*HDA1*/*HOS2*, while In contrast, the meristem regulatory element CAT-box is distributed among multiple genes. This combinatorial *cis*-regulatory architecture—featuring element-specific distributions like DRE restriction to *Fp_HOS3*—demonstrates how functional diversification enables HDACs to integrate light perception, phytohormone signaling, and stress defense networks, collectively orchestrating *F. proliferatum*’s environmental adaptability. While these *in silico* predictions provide valuable hypotheses regarding the potential regulation of *FpHDACs* genes, it is important to note that they do not confirm functional activity. Future work employing promoter-reporter assays will be essential to experimentally validate the role of these predicted *cis*-elements in mediating stress-responsive expression.

### 3.7 Phenotypic analysis of *F. proliferatum* under abiotic stress and qRT-PCR validation of *FpHDACs* genes

**Figure 8:**
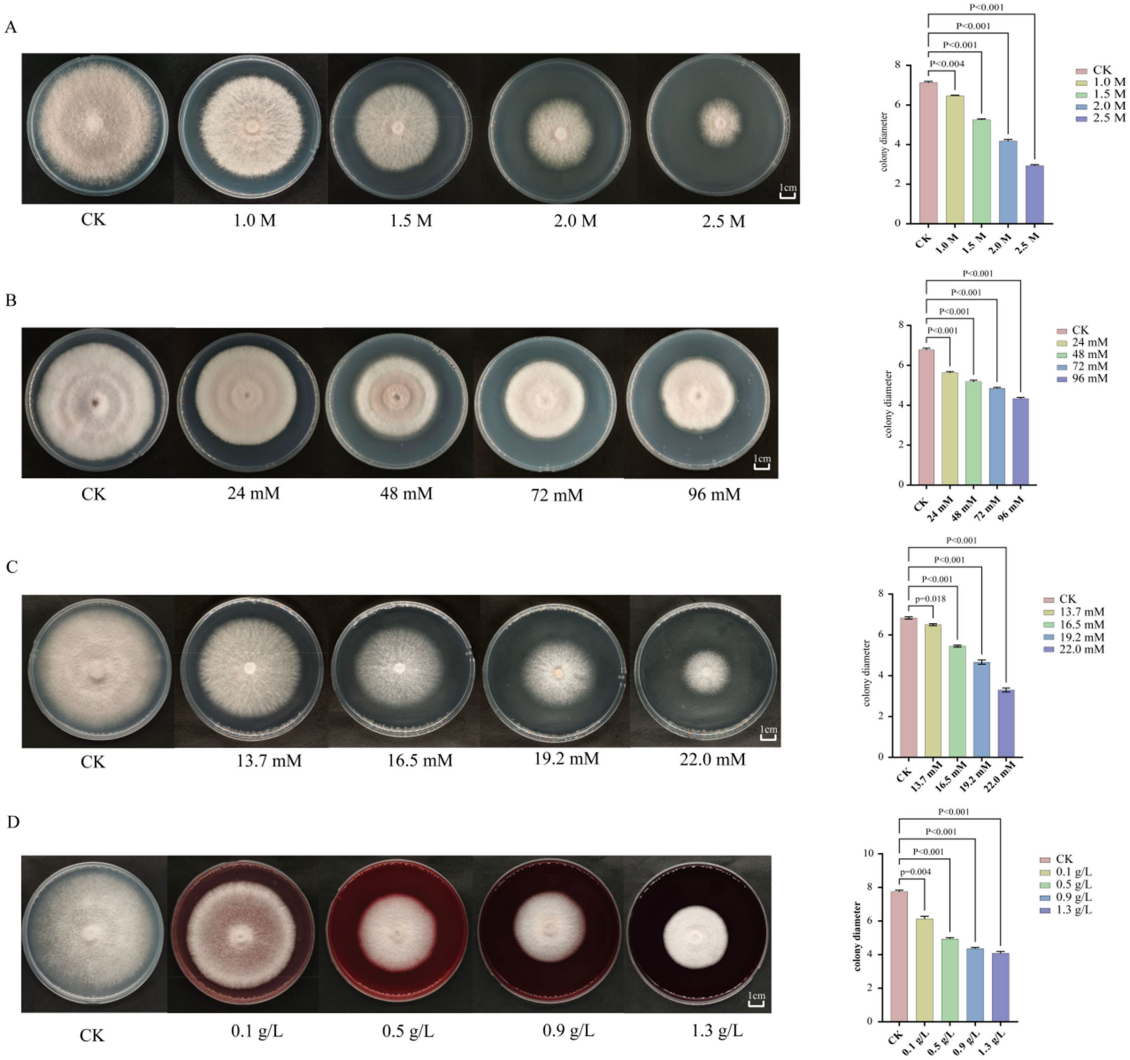
Documents *F. proliferatum*’s phenotypic and growth responses to four abiotic stressors after 7-day incubation. Left panels display representative colony morphologies under escalating stress intensities, while right panels quantify corresponding growth inhibition through colony diameter measurements. Systematic analyses include: **(A)** salt stress (KCl: 1.0 M, 1.5 M, 2.0 M, 2.5 M). **(B)** Oxidative stress (H_2_O_2_: 24 mM, 48 mM, 72 mM, 96 mM). **(C)** Osmotic stress (sorbitol: 13.7 mM, 16.5 mM, 19.2 mM, 22.0 mM). **(D)** Cell wall stress (Congo red: 0.1 g/L, 0.5 g/L, 0.9 g/L, 1.3 g/L). Concentration gradients are denoted in black text with color-coded backgrounds distinguishing stress levels. Statistical analysis was performed using one-way ANOVA followed by Tukey’s HSD *post hoc* test in SPSS. The significance of differences between each stress treatment and the unstressed control is indicated by their respective *p*-values.

**Figure 9:**
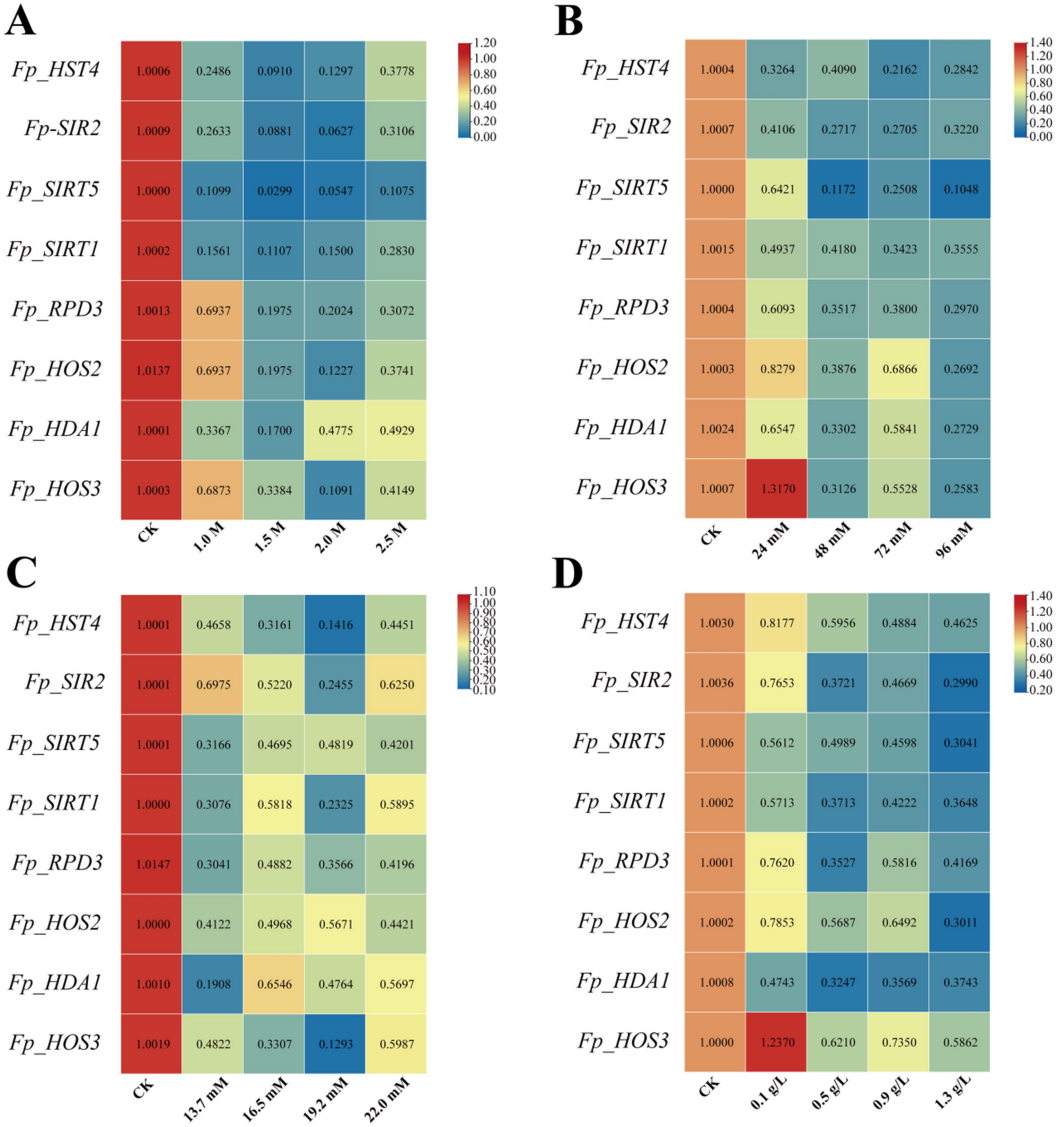
Presents heatmaps profiling transcriptional reprogramming of *FpHDACs* genes under escalating abiotic stress regimes. All data presented in the figure were subjected to statistical analysis using TBtools, with the four stress treatment datasets normalized against the control group (CK) as reference. Each panel documents expression dynamics across four stress intensities: **(A)** salt stress (KCl: 1.0 M, 1.5 M, 2.0 M, 2.5 M). **(B)** Oxidative stress (H_2_O_2_: 24 mM, 48 mM, 72 mM, 96 mM). **(C)** Osmotic stress (sorbitol: 13.7 mM, 16.5 mM, 19.2 mM, 22.0 mM). **(D)** Cell wall perturbation (Congo red: 0.1 g/L, 0.5 g/L, 0.9 g/L, 1.3 g/L). Expression gradients are color-mapped with red indicating significant upregulation and blue denoting downregulation relative to unstressed controls. Numerical expression values appear in black text, while italicized black labels specify treatment concentrations.

To elucidate the role of HDACs genes in the stress tolerance of *F. proliferatum*, we subjected the fungal strain to gradient concentrations of abiotic stressors on PDA plates, including salt stress (KCl), oxidative stress (H_2_O_2_), osmotic stress (sorbitol), and cell wall perturbation (Congo red) (Figure 8). Concurrently, we analyzed the expression profiles of eight *FpHDACs* genes under these four stress conditions using qRT-PCR (Figure 9). After 7 days of incubation, all four stressors significantly inhibited mycelial growth compared to the control, with the inhibition rate increasing in a dose-dependent manner. One-way ANOVA followed with Tukey’s HSD *post hoc* test to compare all concentration treatment groups with the untreated control group, the results revealed significant differences in all stress treatment groups compared to the control group. The colony diameters showed a gradient decrease. The sensitivity of *F. proliferatum* to these stressors followed the order: salt stress > osmotic stress > cell wall perturbation > oxidative stress.

**Figure 10:**
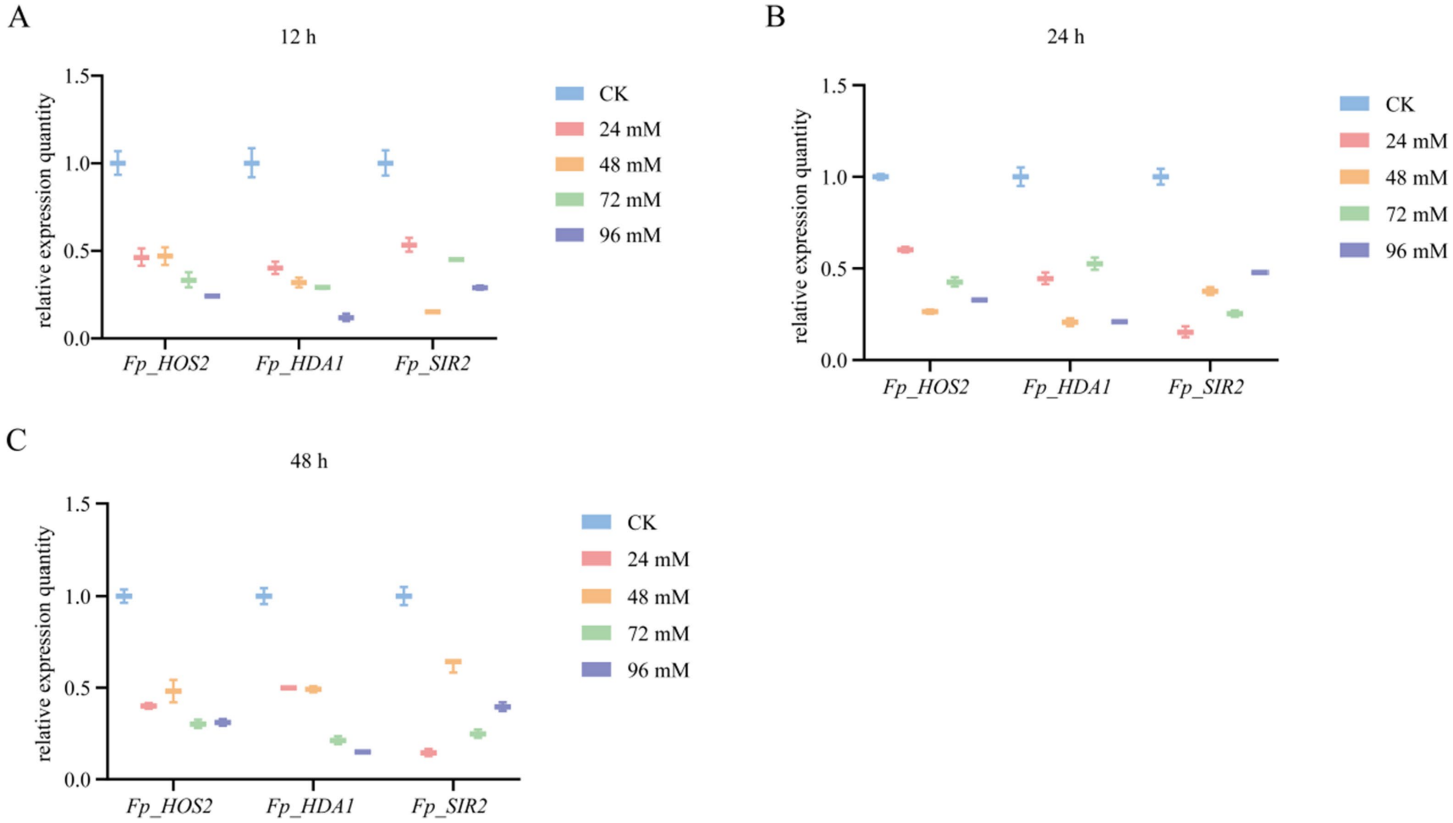
Gene expression profiles following 12-, 24-, and 48-h treatments with varying concentrations of hydrogen peroxide. The expression levels of three genes (*Fp_HOS2*, *Fp_HDA1*, and *Fp_SIR2*) following treatment with varying concentrations of hydrogen peroxide were compared with those of the control group (CK). Statistical analyses were performed using GraphPad Prism 10, visualization was performed using Tukey box plots. The blue box represents CK, the red box denotes treatment with 24 mM hydrogen peroxide, the orange box indicates treatment with 48 mM hydrogen peroxide, the green box signifies treatment with 72 mM hydrogen peroxide, and the purple box corresponds to treatment with 96 mM hydrogen peroxide. **(A)** Changes in gene expression levels following 12-h hydrogen peroxide treatment. **(B)** Gene expression levels following 24-h hydrogen peroxide treatment. **(C)** Changes in gene expression levels following 48-h hydrogen peroxide treatment. The horizontal axis of the graph represents different genes, while the vertical axis indicates relative expression levels, with varying concentrations of hydrogen peroxide denoted in black font.

The phenotypic responses (Figure 8) and qRT-PCR results (Figures 9, 10) collectively demonstrated that all *FpHDACs* genes were responsive to the four abiotic stresses, albeit with distinct regulatory patterns. Under salt stress (KCl), colony diameter decreased progressively with increasing stress intensity (Figure 8A). Although *FpHDACs* expression levels generally declined, they did not follow a strict dose-dependent gradient (Figure 9A). Notably, *FP_HST4* exhibited the most pronounced downregulation but without a stepwise reduction pattern, suggesting differential regulation of *FpHDACs* under salt stress, potentially due to variations in *cis*-regulatory elements or functional divergence. For oxidative stress (H_2_O_2_) and cell wall perturbation (Congo red), colony diameters similarly diminished with rising stress concentrations (Figures 8B,C). qRT-PCR analysis indicated that all *FpHDACs* genes except *FP_HOS3* were downregulated to varying degrees. Strikingly, *Fp_HOS3* displayed transient upregulation prior to decline, implying a biphasic regulatory mechanism specific to these stressors. In osmotic stress (sorbitol), the phenotypic response closely resembled salt stress, with concentration-dependent reductions in colony diameter. All *FpHDACs* genes were downregulated, further supporting their role in osmotic stress adaptation. To further elucidate the temporal dynamics of HDAC gene expression, we quantified transcript levels of *Fp_HOS2* and *Fp_HDA1* (both belonging to the RPD3/HDA1 subfamily) and *Fp_SIR2* (a Sirtuin subfamily member) under H_2_O_2_-induced oxidative stress at 12, 24, and 48 h post-treatment (Figure 10). Distinct temporal expression profiles were observed across these time points. Compared with the control (CK), all three genes showed varying degrees of downregulation under different concentrations of oxidative stress at 12 h (Figure 10A), 24 h (Figure 10B), and 48 h (Figure 10C). This time-dependent modulation highlights the complex involvement of HDACs in the response to prolonged stress.

### 3.8 Pharmacological inhibition of HDACs impairs stress adaptation in *F. proliferatum*

Building on the stress-induced expression profiles of *FpHDACs*, we hypothesized that HDAC activity is critical for fungal stress tolerance. To test this, we perturbed HDAC function using Trichostatin A (TSA, a Class I/II inhibitor) and Nicotinamide (Nic, a sirtuin inhibitor) under four abiotic stress conditions.

**Figure 11:**
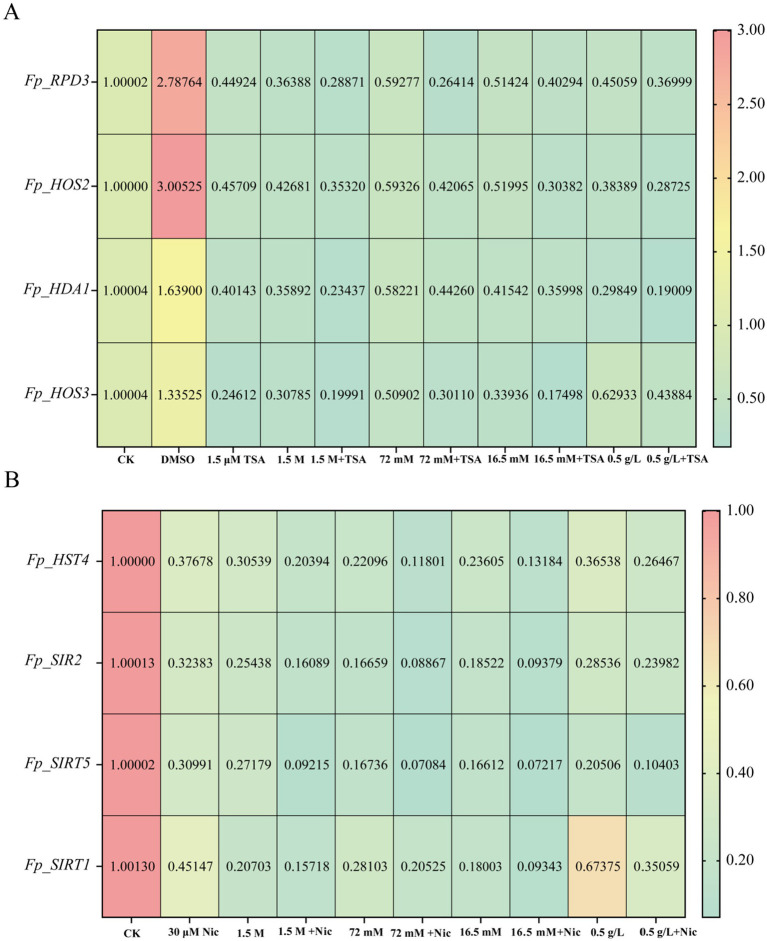
Heat map illustrating alterations in gene expression levels of *F. proliferatum* in response to various abiotic stressors under HDAC inhibitor treatments. Data visualization was performed using GraphPad Prism 10. The four abiotic stressors include salt stress (KCl: 1.5 M), oxidative stress (H_2_O_2_: 72 mM), osmotic stress (sorbitol: 16.5 mM), and cell wall inhibitor (Congo red: 0.5 g/L). Trichostatin A (TSA) and Nicotinamide (Nic) were applied at concentrations of 1.5 μM and 30 μM, respectively, across all stress conditions. **(A)** Effects of the class I/II HDAC inhibitor Trichostatin A (TSA) on gene expression changes in *F. proliferatum* under four abiotic stress conditions. **(B)** Effects of the sirtuin subfamily inhibitor Nicotinamide (Nic) on gene expression levels in *F. proliferatum* under combined abiotic stress and HDAC inhibitor exposure. All treatment groups were normalized to the blank control (CK) and solvent control (DMSO). Gene expression changes are represented by a color scale: red indicates significant upregulation, while green denotes significant downregulation. The *x*-axis represents different treatment groups, and the *y*-axis corresponds to various *FpHDACs* genes. Numerical values of *FpHDACs* genes expression levels are displayed in black within the heat map.

Inhibitor treatments altered fungal colonial growth (Supplementary Figures S1, S2) and markedly affected *FpHDACs* expression. For the RPD3/HDA1 subfamily genes (*Fp_RPD3*, *Fp_HOS2*, *Fp_HDA1*, *Fp_HOS3*), single stressor or 1.5 μM TSA treatments caused downregulation. However, the combined application of TSA with each stressor resulted in a synergistic effect, leading to the most significant suppression of gene expression compared to all control conditions (Figure 11A).

A parallel trend was observed for the SIR2 subfamily genes (*Fp_HST4*, *Fp_SIR2*, *Fp_SIRT5*, *Fp_SIRT1*). Their expression was reduced by individual stresses or 30 μM Nic, and was most severely inhibited under co-treatment with Nic and each stressor (Figure 11B).

These findings demonstrate that HDAC inhibition compromises the stress resilience of *F. proliferatum* and that *FpHDACs* are pharmacologically sensitive to these inhibitors. This provides functional evidence that HDACs play a crucial role in mediating the fungal stress response.

## 4 Discussion

*Fusarium proliferatum*, a globally distributed phytopathogen, severely impacts agricultural productivity by causing destructive root rot in economically vital crops (Guo et al., 2020). Effective disease management remains challenging, necessitating deeper molecular insights into its pathogenicity (Sun et al., 2025). Within fungal epigenetic regulation, histone acetylation dynamics are key regulators of diverse biological processes, including virulence activation and disease-associated secondary metabolite synthesis (Lai et al., 2022). Although substantial evidence establishes histone deacetylases (HDACs) as key regulators of pathogenesis in filamentous fungi (Dubey et al., 2019), their specific functional repertoire in *F. proliferatum* remains unexplored.

Genome-wide analysis identified eight HDACs genes in *F. proliferatum*, phylogenetically resolved into two evolutionarily conserved subfamilies: RPD3/HDA1 and Sirtuin (SIR2) (Figures 1–3). This classification mirrors HDACs organization in *Triticum aestivum* (Jin et al., 2021), demonstrating cross-kingdom conservation of core epigenetic machinery alongside lineage-specific diversification. Analysis of the physicochemical properties of *FpHDACs* members is conducive to understanding their functional differentiation and provides a theoretical basis for subsequent experimental screening of targets, exemplified by *Fp_HOS3* (1,165 aa) and *Fp_SIRT5* (295 aa). The significant differences in sequence length usually reflect the complexity of domain composition; specifically, longer sequences typically contain multiple domains, whereas shorter sequences may only harbor a single core domain (Figure 2). Furthermore, the predicted isoelectric points (pI) of *FpHDACs* exhibit a broad distribution range, ranging from 4.57 to 9.79. Meanwhile, subcellular localization predictions indicate that they are localized to two distinct regions, namely the cytoplasm and the nucleus. These data reveal that most *FpHDACs* likely possess distinct electrochemical properties when adapting to microenvironments that are near-neutral (cytoplasm) or slightly alkaline (nucleus), which in turn affects their activity and localization (Tokmakov et al., 2021). However, this is merely our predictive analysis. In future research, the accuracy of the predicted results needs to be verified through further experiments (such as GFP-tagging).

Domain architecture analysis of *FpHDACs* reveals evolutionary innovations driving functional specialization (Figure 2). Beyond conserved catalytic cores defining RPD3/HDA1 and Sirtuin subfamilies, auxiliary modules enable mechanistic divergence: *Fp_HDA1*’s C-terminal Arb2 domain facilitates homodimerization (Shen et al., 2016)—essential for its deacetylase activity. *Fp_HOS3* uniquely incorporates PHA03247 domain (SMP domain) (Jeyasimman and Saheki, 2020) and PTZ00449 (baculoviral IAP repeat) (Gebreegziabher Amare et al., 2022), suggesting adaptive roles in stress perception or cellular remodeling. *Fp_RPD3*’s LGT domain (Noland et al., 2017) implies unexpected crosstalk between lipoprotein metabolism and epigenetic regulation (Yang et al., 2024). The prediction results of protein–protein interaction networks (Figure 6) show that there are extensive interaction relationships among members of the *FpHDACs* family. This finding is consistent with the analysis results of the *PtrHDAC* gene family in Populus (Li et al., 2024), supporting the general view that HDACs tend to form complexes to exert their functions. In addition, phylogenetic analysis reveals that *FpHDACs* proteins in the same clade exhibit similar distribution patterns of conserved motifs (Figure 3), suggesting that these members may have similar functional characteristics. Gene structure (the number and location of introns and exons) has a significant impact on gene function. We analyzed the intron-exon structure of *FpHDACs* (Figure 5). The results show that all *FpHDACs* genes contain both introns and exons. Among them, *Fp_HDA1* has the largest number of exons and introns, reflecting the potential diversity of transcriptional regulatory mechanisms in this family gene. Notably, no clear correlation was observed between the number of exons and the distribution pattern of conserved motifs. This indicates that the functions of *FpHDACs* genes in some biological processes may be different. For example, in response to abiotic stresses, the expression levels of different *FpHDACs* genes show differential downregulation trends (Figure 9), which also indirectly confirms the functional differences.

*Cis*-acting elements, as key transcriptional regulators, extensively influence various biological processes by participating in the control of gene transcriptional activity. In this study, we analyzed the cis-acting elements in the promoter regions of *FpHDACs* genes. The results showed that these promoter regions contain abundant response elements, most of which are related to the regulation of fungal growth and development as well as stress responses (Figure 7). This finding suggests that *FpHDACs* genes may have multiple potential functional roles in regulating the growth, and development process of *F. proliferatum* and responding to various environmental stresses. Specifically, promoter analysis revealed a high frequency of drought stress response elements (such as MYB) and hormone response elements (such as STRE, MeJA) (Liu et al., 2019). This distribution pattern is similar to the HDAC elements reported in plants such as and *Triticum aestivum* (Li et al., 2022), indicating that *FpHDACs* may respond to adverse growth conditions by integrating multiple environmental signals (such as drought and hormones). The oxidative stress response element (as-1) is distributed in the promoters of all analyzed *FpHDACs* members, which indicates that this gene family plays an important role in the oxidative stress response, and its functional mechanism may involve maintaining cellular redox homeostasis by regulating the expression of reactive oxygen species (ROS)-related genes (Gao et al., 2024). In contrast, specific stress response elements such as dehydration response element (DRE), pathogen/stress response element (W-box), WRE3, and GCN4_motif are specifically enriched only in the promoter regions of some genes (*Fp_HOS3*, *Fp_HOS2*, etc.) (Figure 7). Notably, correlation analysis with the stress-related expression profile data in this study (Figure 9) showed that the expression levels of those *FpHDACs* genes enriched with the above specific elements (DRE, W-box, WRE3, GCN4_motif) exhibited more significant fluctuations under stress conditions (Dhatterwal et al., 2019; Yamaguchi-Shinozaki and Shinozaki, 1994). In summary, compared with HDACs in plant systems, the *FpHDACs* of *F. proliferatum* in this study present a more complex combination of cis-regulatory elements. This difference in regulatory architecture between species may reflect the unique adaptive strategies adopted by phytopathogenic fungi in coping with adverse stresses (Francisco et al., 2019).

Histone deacetylases are evolutionarily conserved mediators of abiotic stress adaptation, particularly oxidative and drought responses (Concepción et al., 2017; Zheng et al., 2016). This paradigm is evidenced by HDA1-dependent oxidative tolerance in and *Penicillium chrysogenum* (Guzman-Chavez et al., 2018) and compromised stress resilience following Sirtuin deletion in *Beauveria bassiana* (Cai et al., 2024). Our phenotypic assays confirm *F. proliferatum*’s acute sensitivity to osmotic, oxidative, and cell wall stressors (Figure 8), establishing its dependence on conserved HDAC-mediated adaptation. Analysis of gene expression patterns serves as a crucial tool for exploring the functions of gene families and their evolutionary relationships. The qRT-PCR results of this study showed that under four abiotic stress conditions, the expression levels of all detected *FpHDACs* genes were inhibited compared with the control group, indicating that *FpHDACs* play a key regulatory role in the response and adaptation of *F. proliferatum* to various abiotic stresses. Notably, under 24 mM hydrogen peroxide stress, the expression levels of all 7 *FpHDACs* genes except *Fp_HOS3* were downregulated to varying degrees. *Fp_HOS3* showed an upregulation trend in the early stage of low-concentration hydrogen peroxide stress, which is similar to the function of the Clr3 protein reported in *Penicillium brasilianum* (Akiyama et al., 2022). Therefore, we speculate that at low concentrations of hydrogen peroxide, ROS may activate the expression of *Fp_HOS3*, which in turn enhances the antioxidant stress capacity of *F. proliferatum* by regulating histone acetylation levels. Similarly, the expression of *Fp_HOS3* was also upregulated under 0.1 g/L Congo red (a cell wall disruptor) stress. Studies have shown that deletion of the hdaA gene in *Aspergillus niger* leads to downregulation of the expression of some cell wall-related genes (Li et al., 2019). In addition, the research has found that the Hog1-MAPK pathway is related to osmotic regulation and participates in the resistance process of antioxidant stress (Wang et al., 2020). From this, we speculate that *Fp_HOS3* may selectively upregulate in response to oxidative stress and cell wall inhibitors by interacting with the Hog1-MAPK pathway.

Histone deacetylases (HDACs) in eukaryotic organisms play critical roles in both physiological and pathological contexts, serving as potential targets for HDAC inhibitors. This study demonstrates that supplementing PDA medium with Trichostatin A (TSA), Nicotinamide (Nic), and four distinct concentrations of abiotic stress agents during *F. proliferatum* cultivation resulted in significant downregulation in the expression levels of eight *FpHDACs* genes. This finding aligns with the observed reduction in HDAC expression following TSA treatment in *Fusarium graminearum* (Amin et al., 2024). Previous studies have demonstrated that HDAC inhibitors such as TSA and Nic can induce the biosynthesis of various secondary metabolites in *Aspergillus awamori* (Lotfy et al., 2021), while the inhibitor SAHA has been shown to upregulate ROS detoxification enzymes (e.g., SOD1, GPx), thereby enhancing cellular resistance to oxidative stress (Wu et al., 2018). Given these findings, our future work will focus on two key objectives: first, to elucidate the mechanisms by which these HDAC inhibitors stimulate secondary metabolite production and upregulate antioxidant enzymes; and second, to explore epigenetically-based strategies for controlling phytopathogenic fungi. Specifically, we will investigate whether HDAC inhibitors can reduce intracellular ROS levels in *Fp_HOS3* by enhancing antioxidant enzyme activity, thereby validating the functional link between HDAC inhibition and the oxidative defense system. Taken together, our qRT-PCR data provide further experimental evidence that *FpHDACs* play a regulatory role in *F. proliferatum*’s resistance to adverse stresses. However, the present study is limited to analyzing gene expression under continuous stress conditions, with no experiments conducted following stress removal. Although HDAC inhibitors and gene knockout approaches were employed to assess expression changes in *FpHDACs* genes, future work should focus on determining whether *FpHDACs* possess functional plasticity and contribute to fungal adaptation. This line of inquiry is motivated by findings in *Fusarium graminearum*, where deletion of genes such as *FgHGG4* enhanced sensitivity to oxidative stress (Park et al., 2023). These findings reflect the evolutionary conservation of HDAC functions in fungal abiotic stress responses, simultaneously demonstrate the diversity of HDAC regulatory mechanisms and adaptive strategies among different fungal species, which will provide new ideas, and theoretical basis for the development of novel control strategies targeting phytopathogenic fungi (Chen et al., 2025; Hu et al., 2024). While the present study provides a comprehensive genomic and transcriptomic framework for the HDAC family in *F. proliferatum* through integrated *in silico* and expression analyses, it is important to acknowledge its limitations. A key limitation of this study is the lack of direct functional validation through gene knockout or CRISPR-mediated silencing of individual *FpHDACs*. This constraint is compounded by our reliance on qRT-PCR to infer changes in acetylation levels indirectly from *FpHDACs* expression. Future work must therefore include the construction of knockout mutants and the use of HDAC-specific inhibitors to establish causal links between individual *FpHDACs* and fungal virulence and stress adaptation. Furthermore, direct assessment of specific histone acetylation marks, for example via western blot analysis, will be essential to unequivocally connect *FpHDACs* expression dynamics to epigenetic remodeling. Finally, as this work was conducted using a single, well-annotated reference strain, subsequent studies should extend these findings to diverse geographical isolates to evaluate the conservation and potential functional divergence of HDACs across different genetic backgrounds.

## 5 Conclusion

This study systematically identifies eight histone deacetylase genes (*FpHDACs*) in *F. proliferatum*, representing the first comprehensive characterization of this epigenetic regulator family in the species. Integrated bioinformatic and functional analyses reveal that *FpHDACs* exhibit evolutionary conservation with homologous HDAC in *Saccharomyces cerevisiae*, *Homo sapiens*, and *Arabidopsis thaliana*, while displaying stronger phylogenetic affinity to fungal orthologs. Structural heterogeneity in gene architecture and conserved motifs suggests functional diversification among family members. Chromosomal distribution patterns indicate evolution through subfunctionalization rather than gene duplication, with all members dispersed across five chromosomes without tandem repeats. Promoter analyses uncovered abundant stress-responsive cis-elements, corroborated by qRT-PCR demonstrating dynamic transcriptional responses to osmotic stress, oxidative challenge, and cell wall disruptors. Moreover, under the suppressive effect of HDAC inhibitors, this dynamic response becomes more pronounced. These findings collectively establish *FpHDACs* as central mediators of environmental adaptation, likely through modulation of ROS homeostasis and cell wall integrity pathways. This work provides foundational insights into fungal epigenetic regulation and identifies novel targets for developing precision antifungal strategies against phytopathogens.

## Data Availability

The datasets presented in this study can be found in online repositories. The names of the repository/repositories and accession number(s) can be found in the article/Supplementary material.

## Footnotes

1
https://www.uniprot.org/

2
https://www.ncbi.nlm.nih.gov/

3
https://www.ebi.ac.uk/interpro/

4
http://cn.expasy.org/tools

5
https://www.genscript.com/wolf-psort.html

6
https://www.chiplot.online/

7
https://meme-suite.org/meme/tools/meme

8
http://gsds.cbi.pku.edu.cn

9
http://fungi.ensembl.org/index.html
