# Supplementary material for: Genome-wide characterization of histone deacetylases in Fusarium proliferatum: phylogeny, structure, and stress responses
Source: Front Microbiol. 2026 Jan 21;16:1692364. doi: 10.3389/fmicb.2025.1692364 (PMC12869994; doi:10.3389/fmicb.2025.1692364)
Supplement: Supplementary file 1 [file Data_Sheet_1.docx]

**Genome-wide Characterization of Histone Deacetylases in *Fusarium proliferatum*: Phylogeny, Structure, and Stress Responses**

**Abstract**

*Fusarium proliferatum*, a globally distributed phytopathogen causing destructive root rot in economically vital crops, employs sophisticated epigenetic mechanisms for environmental adaptation. Our genome-wide characterization identified eight histone deacetylase (*FpHDACs*) genes phylogenetically classified into RPD3/HDA1 and Sirtuin subfamilies. Comprehensive genomic characterization revealed two distinctive features: expanded domain architectures exemplified by the Arb2 domain within *Fp_HDA1*, and bipartite subcellular localization mediated through electrostatic partitioning—where most *FpHDACs* reside cytoplasmically under neutral pH but undergo nuclear translocation in alkaline environments. Evolutionary diversification occurred principally via subfunctionalization rather than gene duplication, evidenced by non-clustered chromosomal distribution (8 genes across 5 chromosomes), divergent gene architectures in intron-exon organization and CDS lengths, and promoter *cis*-element enrichment featuring combinatorial stress-responsive signatures, most notably the dehydration-responsive DRE motifs exclusive to *Fp_HOS3*. Expression profiling demonstrated a conserved global repression of *FpHDACs* under abiotic stresses, crucially counterbalanced by context-dependent induction of *Fp_HOS3* during oxidative and cell wall stress. This specialized isoform functions as a compensatory epigenetic modulator, fine-tuning stress responses through targeted histone modification. Collectively, these findings unveil dual conservation-innovation strategies in fungal stress epigenetics, positioning stress-adapted HDACs complexes like *Fp_HOS3* as strategic targets for next-generation antifungals via epigenetic interference.

**Key words:** Histone deacetylases (HDACs); *Fusarium proliferatum*; Gene family evolution; Abiotic stress response; Epigenetic regulation.

Supplementary Table 1. Primers for RT-qPCR used in this paper

| Primer Name | Forward primer(5'to 3') | Reverse primer(5'to 3') |
| --- | --- | --- |
| *FP_HST4* | AGCCCGAAGATGAACTCACG | CCTGCACCAGCAATGACAAC |
| *FP_HDA1* | TTGCCGGAAGGTTCTCATCC | CCGCATTTGTCGATTCCACC |
| *FP_HOS3* | CTGCTCCCATCTCTTCGGAC | CGGGATCTCACTAAGACGGC |
| *FP_SIRT1* | CCAAGCTTCTGCGTCACAAC | TCAGGAATGCCAAGCGATGT |
| *FP_SIRT5* | GTCATATGTGCTTGCGCTCG | AATCGCAGTTGGTGCACTTG |
| *FP_RPD3* | GGTGATGTTGGCATGGAGGA | GGCTCCTTCGTAGCGGTATC |
| *FP_SIR2* | CAAGCGCGTCTGAATGAAGG | TCGACCTGGATTGTGTCTGC |
| *FP_HOS2* | GAGCCACGAGTGAATCGGAA | TCCTTCAGCCGTTCCTCAAC |
| QTUB | TTCTGCTGTCATGTCCGGTGT | TCAGAGGAGCAAAGCCAACCA |

Supplementary Table 2. full inventory cis-acting element in *FpHDACs* genes

| cis-acting element | *Fp_HST4* | *Fp_SIR2* | *Fp_SIRT5* | *Fp_SIRT1* | *Fp_HOS3* | *Fp_HDA1* | *Fp_HOS2* | *Fp_RPD3* |
| --- | --- | --- | --- | --- | --- | --- | --- | --- |
| WRE3 | 1 | 0 | 1 | 1 | 1 | 0 | 1 | 0 |
| W-box | 1 | 0 | 0 | 2 | 0 | 3 | 1 | 1 |
| TGA-element | 0 | 3 | 1 | 0 | 0 | 2 | 1 | 2 |
| TGACG-motif | 3 | 7 | 2 | 2 | 2 | 5 | 5 | 6 |
| TCT-motif | 0 | 0 | 3 | 0 | 0 | 1 | 2 | 0 |
| TCCC-motif | 0 | 0 | 0 | 0 | 0 | 0 | 0 | 2 |
| TATA-box | 5 | 8 | 34 | 18 | 14 | 9 | 2 | 2 |
| STRE | 2 | 3 | 2 | 7 | 7 | 2 | 2 | 1 |
| Sp1 | 0 | 0 | 0 | 0 | 0 | 0 | 0 | 2 |
| RY-element | 0 | 0 | 0 | 0 | 0 | 0 | 0 | 1 |
| re2f-1 | 1 | 0 | 0 | 0 | 0 | 0 | 0 | 0 |
| P-box | 0 | 1 | 0 | 0 | 0 | 0 | 0 | 0 |
| O2-site | 0 | 0 | 0 | 1 | 0 | 0 | 0 | 1 |
| MYC | 3 | 2 | 4 | 4 | 4 | 1 | 5 | 3 |
| MYB-binding site | 0 | 0 | 0 | 0 | 2 | 2 | 0 | 2 |
| MYB | 2 | 3 | 6 | 8 | 10 | 7 | 3 | 4 |
| MRE | 0 | 0 | 1 | 1 | 0 | 0 | 0 | 0 |
| MBS | 1 | 2 | 1 | 3 | 3 | 1 | 1 | 0 |

Continued supplementary Table 2. full inventory cis-acting element in *FpHDACs* genes

| cis-acting element | *Fp_HST4* | *Fp_SIR2* | *Fp_SIRT5* | *Fp_SIRT1* | *Fp_HOS3* | *Fp_HDA1* | *Fp_HOS2* | *Fp_RPD3* |
| --- | --- | --- | --- | --- | --- | --- | --- | --- |
| I-box | 1 | 0 | 0 | 0 | 1 | 1 | 1 | 0 |
| GT1-motif | 1 | 0 | 1 | 1 | 1 | 2 | 1 | 0 |
| GCN4_motif | 0 | 1 | 0 | 0 | 0 | 1 | 1 | 0 |
| GC-motif | 2 | 1 | 1 | 0 | 0 | 1 | 0 | 1 |
| G-box | 5 | 5 | 1 | 0 | 3 | 3 | 0 | 5 |
| GATA-motif | 1 | 1 | 1 | 1 | 0 | 0 | 0 | 2 |
| ERE | 0 | 0 | 1 | 0 | 2 | 1 | 0 | 0 |
| DRE1 | 0 | 1 | 0 | 0 | 0 | 1 | 0 | 0 |
| DRE-core | 0 | 2 | 0 | 1 | 2 | 1 | 1 | 0 |
| DRE | 0 | 0 | 0 | 0 | 1 | 0 | 0 | 0 |
| CGTCA-motif | 3 | 7 | 1 | 2 | 2 | 5 | 5 | 6 |
| CCGTCC-box | 2 | 0 | 0 | 0 | 0 | 0 | 1 | 1 |
| CCGTCC motif | 2 | 0 | 0 | 0 | 0 | 0 | 1 | 1 |
| CCAAT-box | 0 | 0 | 0 | 0 | 2 | 0 | 0 | 1 |
| CAT-box | 2 | 4 | 3 | 1 | 1 | 0 | 1 | 0 |
| CARE | 0 | 0 | 1 | 0 | 0 | 0 | 0 | 1 |
| CAAT-box | 31 | 21 | 19 | 22 | 21 | 19 | 14 | 24 |
| box S | 1 | 0 | 0 | 0 | 0 | 1 | 0 | 0 |
| Box 4 | 0 | 1 | 1 | 1 | 0 | 0 | 0 | 2 |

Continued supplementary Table 2. full inventory cis-acting element in *FpHDACs* genes

| cis-acting element | *Fp_HST4* | *Fp_SIR2* | *Fp_SIRT5* | *Fp_SIRT1* | *Fp_HOS3* | *Fp_HDA1* | *Fp_HOS2* | *Fp_RPD3* |
| --- | --- | --- | --- | --- | --- | --- | --- | --- |
| AT~TATA-box | 1 | 0 | 5 | 2 | 2 | 0 | 0 | 0 |
| as-1 | 3 | 4 | 2 | 2 | 2 | 5 | 5 | 6 |
| ARE | 2 | 2 | 0 | 1 | 1 | 5 | 0 | 0 |
| AE-box | 1 | 0 | 0 | 0 | 1 | 0 | 1 | 0 |
| ABRE | 6 | 4 | 1 | 0 | 2 | 2 | 0 | 4 |
| A-box | 2 | 0 | 0 | 0 | 0 | 0 | 1 | 1 |


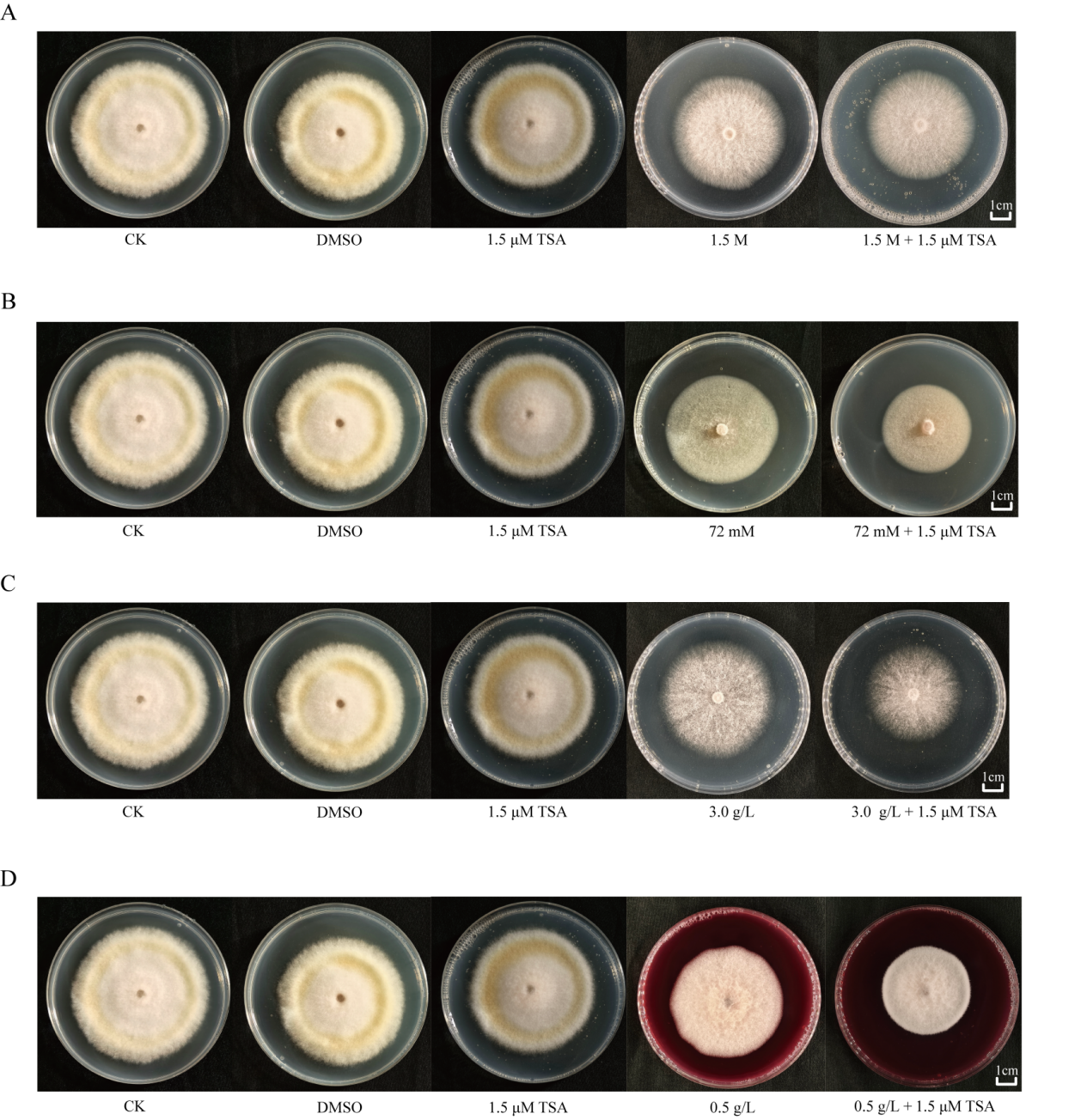
FIGURE S1

Colony morphology of *F. proliferatum* after 7 days of growth under Trichostatin A (TSA) and four abiotic stress conditions. CK and DMSO were used as blank control and solvent control, respectively, with black labels indicating treatment groups at different concentrations. (A) Colony morphology under salt stress (KCl: 1.5 M) and 1.5 M KCl + 1.5 μM TSA. (B) Colony morphology under oxidative stress (H₂O₂: 72 mM) and 72 mM H₂O₂ + 1.5 μM TSA. (C) Colony variation under osmotic stress (sorbitol: 3.0 g/L) and 3.0 g/L sorbitol + 1.5 μM TSA. (D) Effect of cell wall inhibitor (Congo red: 0.5 g/L) and 0.5 g/L Congo red + 1.5 μM TSA on the growth of *F. proliferatum*.


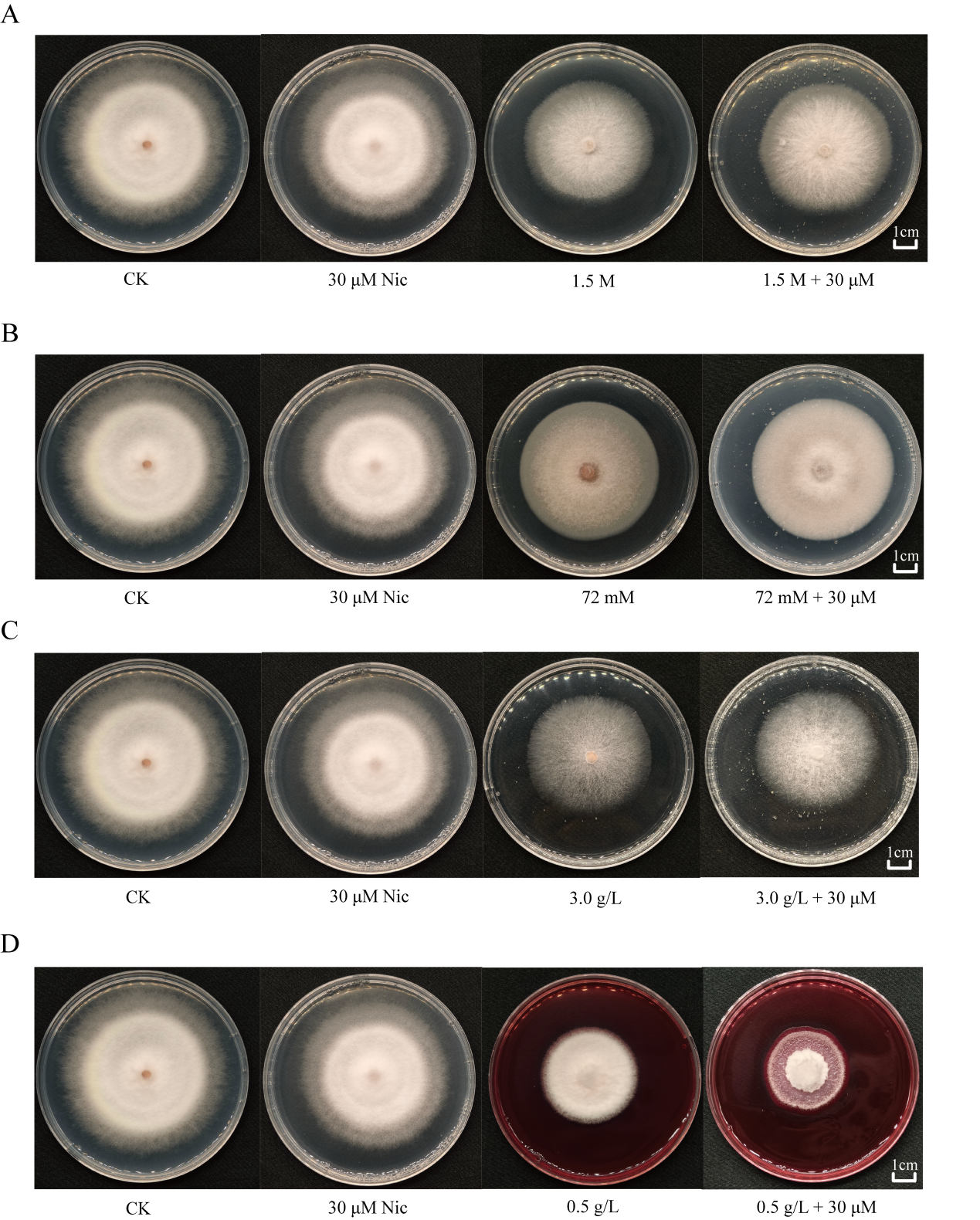
Figure S2

Colony morphology of *F. proliferatum* after 7 days of growth under Nicotinamide (Nic) and four abiotic stress conditions. CK was used as blank control, with black labels indicating treatment groups at different concentrations. (A) Colony morphology under salt stress (KCl: 1.5 M) and 1.5 M KCl + 30 μM Nic. (B) Colony morphology under oxidative stress (H₂O₂: 72 mM) and 72 mM H₂O₂ + 30 μM Nic. (C) Colony variation under osmotic stress (sorbitol: 3.0 g/L) and 3.0 g/L sorbitol + 30 μM Nic. (D) Effect of cell wall inhibitor (Congo red: 0.5 g/L) and 0.5 g/L Congo red + 30μM Nic on the growth of *F. proliferatum*.
